# Sodium benzoate attenuates 2,8-dihydroxyadenine nephropathy by inhibiting monocyte/macrophage TNF-α expression

**DOI:** 10.1038/s41598-023-30056-6

**Published:** 2023-02-27

**Authors:** Yoichi Oshima, Shu Wakino, Takeshi Kanda, Takaya Tajima, Tomoaki Itoh, Kiyotaka Uchiyama, Keiko Yoshimoto, Jumpei Sasabe, Masato Yasui, Hiroshi Itoh

**Affiliations:** 1 Department of Internal Medicine, Keio University School of Medicine, Tokyo, Japan; 2 Department of Nephrology, Tokushima University School of Medicine, Tokushima, Japan; 3 Department of Pharmacology, Keio University School of Medicine, Tokyo, Japan

**Keywords:** Interstitial nephritis, Molecular medicine

## Abstract

Sodium benzoate (SB), a known D-amino acid oxidase (DAO) enzyme inhibitor, has an anti-inflammatory effect, although its role in renal damage has not been explored. 2,8-dihydroxyadenine crystal induced chronic kidney disease, in which TNF-α is involved in the pathogenesis, was established by oral adenine administration in C57BL/6JJcl mice (AdCKD) with or without SB to investigate its renal protective effects. SB significantly attenuated AdCKD by decreasing serum creatinine and urea nitrogen levels, and kidney interstitial fibrosis and tubular atrophy scores. The survival of AdCKD mice improved 2.6-fold by SB administration. SB significantly decreased the number of infiltrating macrophages observed in the positive F4/80 immunohistochemistry area and reduced the expression of macrophage markers and inflammatory genes, including TNF-α, in the kidneys of AdCKD. Human THP-1 cells stimulated with either lipopolysaccharide or TNF-α showed increased expression of inflammatory genes, although this was significantly reduced by SB, confirming the anti-inflammatory effects of SB. SB exhibited renal protective effects in AdCKD in DAO enzyme deficient mice, suggesting that anti-inflammatory effect of SB was independent of DAO enzyme activity. Moreover, binding to motif DNA sequence, protein level, and mRNA level of NF-κB RelB were significantly inhibited by SB in AdCKD kidneys and lipopolysaccharide treated THP-1 cells, respectively. We report that anti-inflammatory property of SB is independent of DAO enzymatic activity and is associated with down regulated NF-κB RelB as well as its downstream inflammatory genes such as TNF-α in AdCKD.

## Introduction

Chronic kidney disease (CKD) is a health problem affecting over 10% of the global population^1^. CKD results in end-stage renal disease that requires dialysis. Patients with CKD who are on dialysis are susceptible to multiple comorbidities, including life-threatening cardiovascular diseases^2,3^ and cancer^4^. Therefore, effective treatment of CKD is essential.

Crystal nephropathy is a form of CKD characterized by the crystallization of uric acid, calcium oxalate, calcium phosphate, and adenine^5^. These crystals induce renal inflammation in an auto-amplification manner^5^. Adenine-induced 2,8-dihydroxyadenine (2,8-DHA) crystal nephropathy is an animal CKD model of a human autosomal recessive genetic disorder caused by adenine phosphoribosyl transferase (APRT) deficiency^6^. Adenine-containing chow, 2,8-DHA nephropathy, or adenine-induced CKD (AdCKD) induced the deposition of crystals in renal tubules, causing inflammation and tubular injury that was followed by interstitial fibrosis and collagen deposition in mice^7^. The AdCKD model mimics human CKD features, including kidney atrophy and fibrosis, elevated urea nitrogen and creatinine levels, anemia, cardiovascular calcifications, cardiac hypertrophy, and elevated blood pressure^8^. Several studies have shown that inflammation and macrophage activation are involved in the pathogenesis of AdCKD. Deletion of *Tnfr1,* the gene encoding a TNF-α receptor, ameliorated disease progression in AdCKD mice^7^. Inhibition of NF-κB, a molecule involved in the TNFα/Tnfr1 cascade, by pyrrolidine dithiocarbamate also attenuated AdCKD progression^9^. Ozone therapy attenuated AdCKD by decreasing the expression of toll-like receptor 4 (TLR4)^10^, a lipopolysaccharide (LPS) receptor. Endoplasmic reticulum (ER) stress was also elevated in the kidneys of AdCKD mice, although this was reduced by the fatty acid receptor GPR40 agonist and aggravated in GPR40 knockout mice^11^. Since GPR40 signaling reduces ER stress^12^ and ER stress and NF-κB have an integrated crosstalk^13^, the inflammatory pathway induced by NF-κB signaling is also important as a downstream inducer of ER stress. AdCKD was also attenuated in CCL3 and CCR5 knockout mice by the monocyte/macrophage depleting agent, clodronate liposomes, suggesting that monocyte/macrophage chemotaxis contributes to the pathogenesis^14^.

Sodium benzoate (SB) is a salt of benzoic acid, a cinnamon-derived metabolite used as a food and cosmetic additive^15^. SB is also a competitive inhibitor of d-amino acid oxidase (DAO), a molecule found in the brain, liver, and kidneys in humans and oxidizes d-amino acids to α-keto acids and hydrogen peroxide^16^. Additionally, SB has been reported to possess anti-inflammatory property^17,18^, although whether the effect is associated with DAO or not have not been determined.

Therefore, we have investigated whether SB could exert anti-inflammatory effects in AdCKD or lipopolysaccharide treated THP-1 cells. We also describe for the first time the underlying mechanism of anti-inflammation leading to alleviated AdCKD.

## Results

### Adenine-induced nephropathy is attenuated by oral administration of sodium benzoate

**Figure 1:**
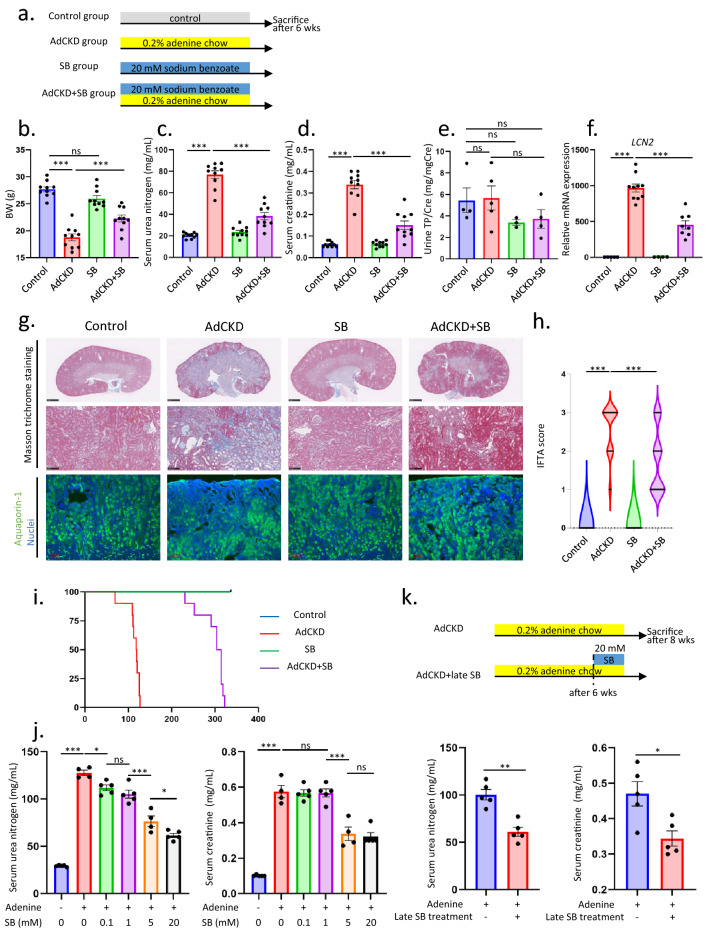
SB attenuates adenine induced CKD. (**a**) Seven-week-old mice were divided into four groups: control, adenine CKD (AdCKD), SB, and AdCKD + SB. The relevant groups were treated with SB in the last two weeks during eight weeks of adenine intake and the mice were sacrificed six weeks later. Changes in body weight (**b**), plasma urea nitrogen (**c**), plasma creatinine (**d**), and urine total protein creatinine ratios (**e**) were examined (n = 10 each). (**f**) *Lcn2* mRNA levels in kidney cortex homogenates were measured using RT-PCR (n = 4–10). (**g**) Representative images of masson-trichrome staining and aquaporin-1 immunofluorescent staining. (**h**) Violin plot showing IFTA scores of randomly acquired images (n = 50 each). (**i**) Survival curves of all groups of mice were examined (n = 10 each). (**j**) Plasma urea nitrogen and creatinine levels in AdCKD mice treated with different concentrations of SB (n = 4–5). For urea nitrogen, *p*-value for respective pair was as follows. Control vs AdCKD, *p* < 0.0001; AdCKD versus AdCKD 0.1 mM SB, *p* = 0.0318; AdCKD 1 mM SB vs AdCKD 5 mM SB, *p* < 0.0001; AdCKD 5 mM SB versus AdCKD 20 mM SB, *p* = 0.0459. For creatinine, *p*-value for respective pair was as follows. Control versus AdCKD, *p* < 0.0001; AdCKD 1 mM SB versus AdCKD 5 mM SB, *p* < 0.0001. (**k**) The effectiveness of late-stage SB administration was confirmed in the AdCKD model by measuring plasma urea nitrogen and creatinine levels (n = 5 each). *p < 0.05; **p < 0.01; ***p < 0.001; ns, not significant. Each bar represents mean ± SEM. AdCKD, adenine induced chronic kidney disease; SB, sodium benzoate; TP/Cre, total protein creatinine ratio; LCN2, lipocalin-2; IFTA, interstitial fibrosis and tubular atrophy; CKD, chronic kidney disease.

We divided mice into four groups; control group, adenine-induced CKD (AdCKD) group, sodium benzoate group, and sodium benzoate treated AdCKD group (Fig. 1a). Body weight decreased in AdCKD mice compared to control mice and was improved in the AdCKD + SB mice compared to AdCKD mice (Fig. 1b). Body weights were similar between the control mice and SB mice. Plasma urea nitrogen and creatinine, which is the hallmark of kidney function, was increased in the AdCKD compared to control, whereas theses were significantly lower in the AdCKD + SB mice (Fig. 1c,d). Proteinuria was similar among the four groups (Fig. 1e), whereas renal tubule damage was aggravated in AdCKD compared to control mice, which was significantly attenuated in the AdCKD + SB mice as shown in tubule damage marker lipocalin-2 (LCN-2), also known as the neutrophil gelatinase-associated lipocalin, mRNA levels (Fig. 1f) and interstitial fibrosis tubular atrophy score (Fig. 1g,h). The elevation of LCN-2 mRNA level in AdCKD was compatible with previous report^7^. AdCKD mice did not have increased proteinuria compared to controls, as shown in Fig. 1e, which was compatible with previous report^19^. This is likely explained by the C57BL/6 strain's known resistance towards development of proteinuria in combination with the tubulointerstitial nature of the renal damage^20^, as reviewed previously^19^. Aquaporin-1 immunofluorescence was lower in AdCKD kidneys, indicating loss of proximal tubular cells in the kidneys of AdCKD mice which was improved in AdCKD + SB mice (Fig. 1g). The average survival period in the AdCKD group was significantly shortened to 113 days compared to control group. However, the survival was significantly improved more than 2.6-fold in the AdCKD + SB group of 296 days compared to AdCKD group (Fig. 1i).

Treatment with 1 mM of SB had no therapeutic effect on kidney function, based on evaluation of creatinine concentrations in plasma, whereas 5 mM of SB exerted a therapeutic effect, showing a dose-dependent response (Fig. 1j). For plasma urea nitrogen levels, 0.1 mM of SB in AdCKD exerted a significant decrease compared to AdCKD alone (Fig. 1j). Urea nitrogen level improved also showing a dose responsiveness as depicted in Fig. 1j. Therefore, a dose dependent improvement of kidney function was confirmed by SB in AdCKD.

As shown in Fig. 1k, we established AdCKD by administering adenine for six weeks prior to SB addition to seek the SB renal protection is seen in already damaged kidneys. The therapeutic effect was still observed, as confirmed by reduced plasma urea nitrogen and creatinine levels (Fig. 1k).

### SB attenuates AdCKD regardless of DAO enzyme activity

**Figure 2:**
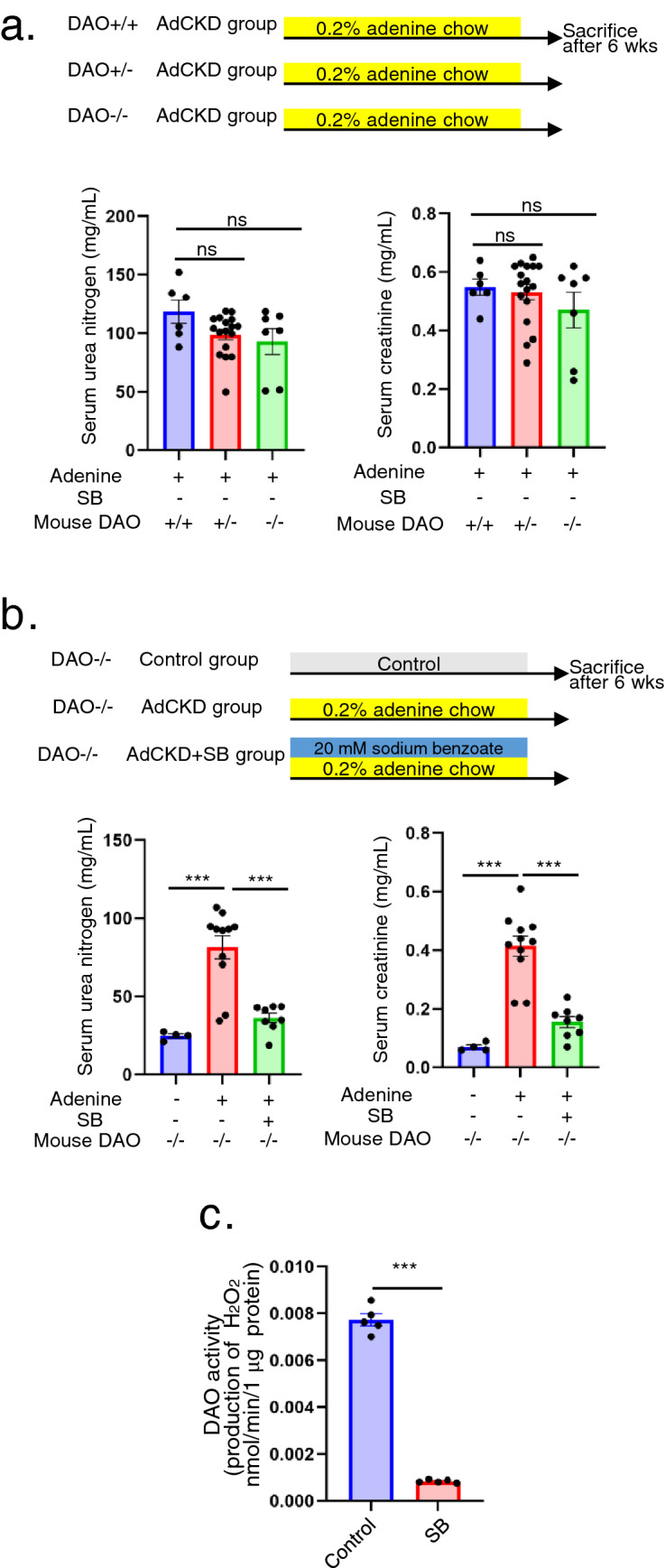
AdCKD is attenuated by SB regardless of DAO enzymatic activity. (**a**) AdCKD was induced in DAO + / + , DAO + / − , and DAO-/- littermates and plasma urea nitrogen and creatinine concentrations compared (n = 6–17). (**b**) DAO − / −  mice were divided into control, AdCKD, and AdCKD + SB groups. SB significantly reduced plasma urea nitrogen and creatinine concentrations (n = 4–11). (**c**) DAO activity assay of normal kidney homogenate shows SB significantly reduced DAO activity (n = 5 each). ***p < 0.001; ns, not significant. Each bar represents mean ± SEM. AdCKD, adenine induced chronic kidney disease; DAO, D-amino acid oxidase.

Since SB is a well-known inhibitor of DAO enzyme activity, we examined whether DAO activity in mice plays a role in AdCKD using the DAO-deficient mouse with C57BL6 background (DAO − / − )^21^. We obtained littermates of wild-type DAO (DAO + / +), heterozygous deficient DAO(+ / − ), and homozygous deficient DAO( − / − ) mice. The littermates were fed chow containing 0.2% adenine. However, no significant differences were observed in the plasma concentrations of urea nitrogen and creatinine between the study groups (Fig. 2a). To examine whether SB exerts a therapeutic effect in AdCKD DAO( − / − ) mice, the mice were divided into three groups: DAO( − / − ) control, AdCKD-induced DAO( − / − ), and AdCKD-induced DAO( − / − ) mice treated with SB. We observed a significant reduction in serum urea nitrogen and creatinine levels in AdCKD-induced DAO( − / − ) mice treated with SB compared with that of AdCKD-induced DAO( − / − ) mice that were not treated with SB (Fig. 2b). We also confirmed the direct inhibition of normal kidney DAO activity by SB (Fig. 2c). Collectively, these results suggest that SB exerts a therapeutic effect regardless of DAO enzymatic activity in AdCKD mice.

### SB decreases macrophage infiltration and the expression of inflammatory genes in AdCKD mice

**Figure 3:**
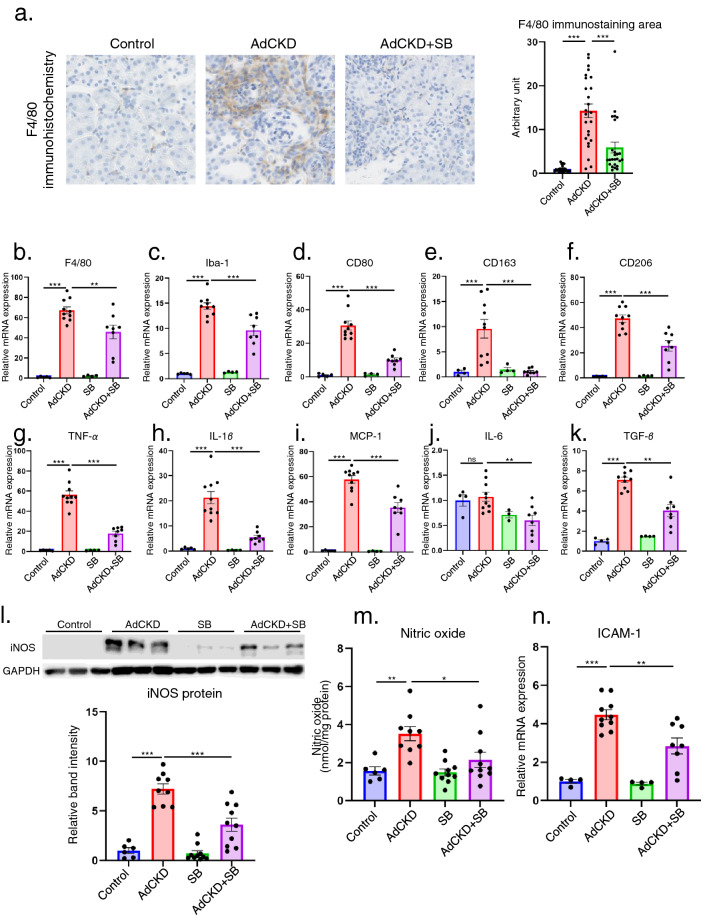
SB attenuated macrophage infiltration and various macrophage markers. (**a**) Plot of F4/80 immunohistochemistry staining areas of randomly acquired high-power field images. The bar graph in the right panel shows the quantifications of the stained area (n = 25 each). SB reduced relative expression of pan-macrophage markers (**b**) *F4/80* and (**c**) *Iba-1*, M1 macrophage markers (**d**) *CD80*, (**g**) *TNF-α*, (**h**)* IL-1β*, (**i**) *MCP-1*, and (**j**) *IL-6*, M2 macrophage markers (**e**) *CD163*, (**f**) *CD206*, and (**k**) *TGF-β*, and contact molecule (**n**) *ICAM-1* in the kidney (n = 3–10). *HPRT* was used as an internal control. Western blots and nitric oxide assays, respectively, showed that the abundance of the M1 macrophage marker (**l**) iNOS and (**m**) nitric oxide level was decreased following SB treatment (n = 6–10). *p < 0.05; **p < 0.01; ***p < 0.001; ns, not significant. Each bar represents mean ± SEM. Iba-1, ionized calcium binding adaptor molecule 1; TNF-α, tumor necrosis factor α; IL-1β, interleukin-1 beta; MCP-1, monocyte chemoattractant protein-1; iNOS, inducible nitric oxide synthase; ICAM-1, intercellular adhesion molecule-1; HPRT, hypoxanthine guanine phosphoribosyl transferase; SB, sodium benzoate.

Macrophage infiltration was elevated in the kidneys of AdCKD mice, consistent with previous data^14^. This increase in macrophage infiltration was significantly reduced by SB, based on F4/80 immunohistochemistry results (Fig. 3a). RT-PCR of kidney cortex samples showed that the expression of macrophage markers such as F4/80, Iba-1, CD 80, CD163, and CD206 was upregulated in AdCKD mice compared with that in the control mice, and this expression was ameliorated by SB (Fig. 3b–f). F4/80 and Iba-1 are pan-macrophage markers, CD 80 is a type 1 (M1) macrophage marker, and CD163 and CD206 are type 2 (M2) macrophage markers. Additionally, the expression of inflammatory M1 macrophage cytokines, including *TNF-α, IL-1β, MCP-1*, and *IL-6*, and the M2 cytokine, *TGF-β*, was upregulated in the kidneys of AdCKD mice compared with that in the control mice. However, the levels of these markers were significantly decreased following SB treatment (Fig. 3g–k). Inducible NOS (iNOS or NOS2) is a hallmark M1 macrophage molecule that produces nitric oxide (NO)^22^. Expression levels of iNOS protein (Fig. 3l) and tissue concentrations of NO (Fig. 3m) were significantly elevated in AdCKD-induced kidneys compared with those in the control kidneys, although this was significantly reduced following SB treatment. These data indicate that both M1 and M2 macrophage infiltration in the kidneys of AdCKD mice was reduced by SB treatment. Finally, the expression of ICAM-1, which is important for monocyte or macrophage tissue adhesion, increased in the kidneys of AdCKD mice, but was attenuated by SB treatment (Fig. 3n).

### SB reduces MAPK and NF-κB p65 signals in the kidneys of AdCKD mice

**Figure 4:**
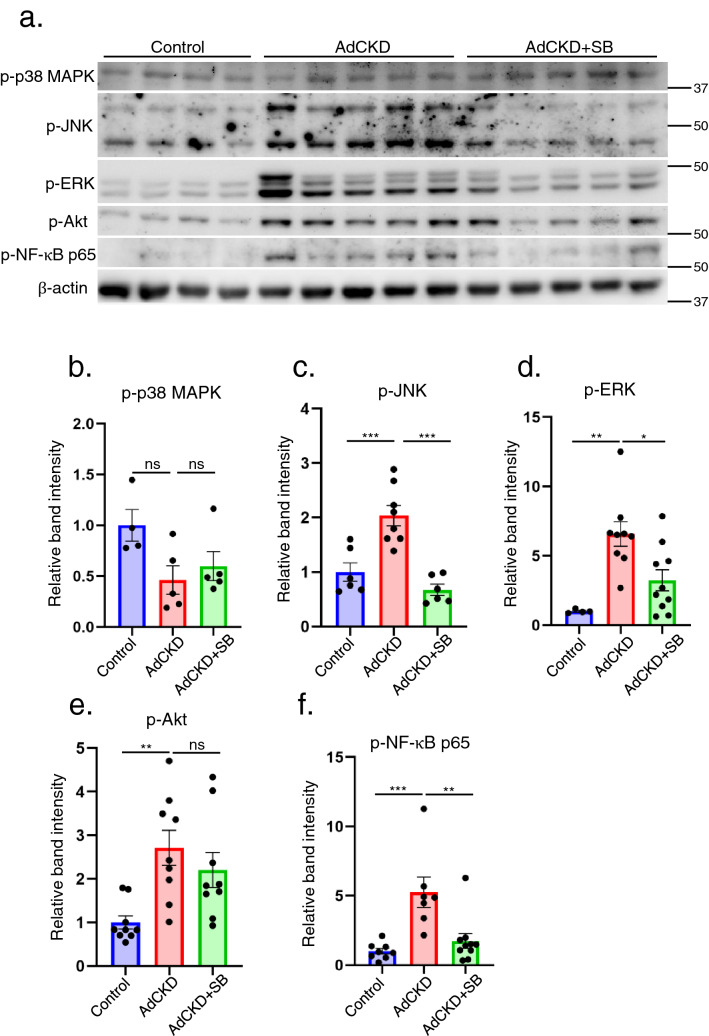
SB reduces the phosphorylation of JNK, ERK, and NF-κB p65 in the kidneys of AdCKD mice. (**a**) Immunoblots of the respective groups are shown. Band intensities of (**b**) phopho-p38 MAPK (p-p38 MAPK), (**c**) phospho-JNK (p-JNK), (**d**) phospho-ERK (p-ERK), (**e**) phospho-Akt (p-Akt), and (**f**) phospho-NF-κB p65 (p-NF-κB p65) were quantified and adjusted to the intensities of the internal control molecule β-actin (n = 4–10). Mice in the AdCKD + SB group showed decreased levels of p-JNK, p-ERK, and p-NF-κB p65 compared with mice in the AdCKD group. *p < 0.05; **p < 0.01; ***p < 0.001; ns, not significant. Each bar represents mean ± SEM. SB, sodium benzoate.

To investigate the involvement of the signal transduction pathway in the kidneys of SB-treated AdCKD mice, we examined the activation of the MAP kinase (MAPK), PI3 kinase/Akt, and NF-κB p65 pathways, since these pathways are closely associated with proinflammatory cytokine signal transduction^23–25^ (Fig. 4a). The expression of phospho-JNK, phospho-ERK, phospho-Akt, and phospho-NF-κB p65 was upregulated in the kidneys of AdCKD mice, whereas phospho-p38 levels remained constant. The upregulated expression of phospho-JNK, phospho-ERK, and phospho-NF-κB p65 was significantly attenuated following SB treatment, whereas that of phospho-Akt was unaltered (Fig. 4b–f). These changes were consistent with the reduced expression of inflammatory molecules such as *TNF-α, IL-1β*, and *MCP-1*, since these molecules have downstream mediators such as MAPK and NF-κB signals^25,26^.

### SB suppresses expression of LPS or TNF-α-induced inflammatory genes in human THP-1 monocytes

**Figure 5:**
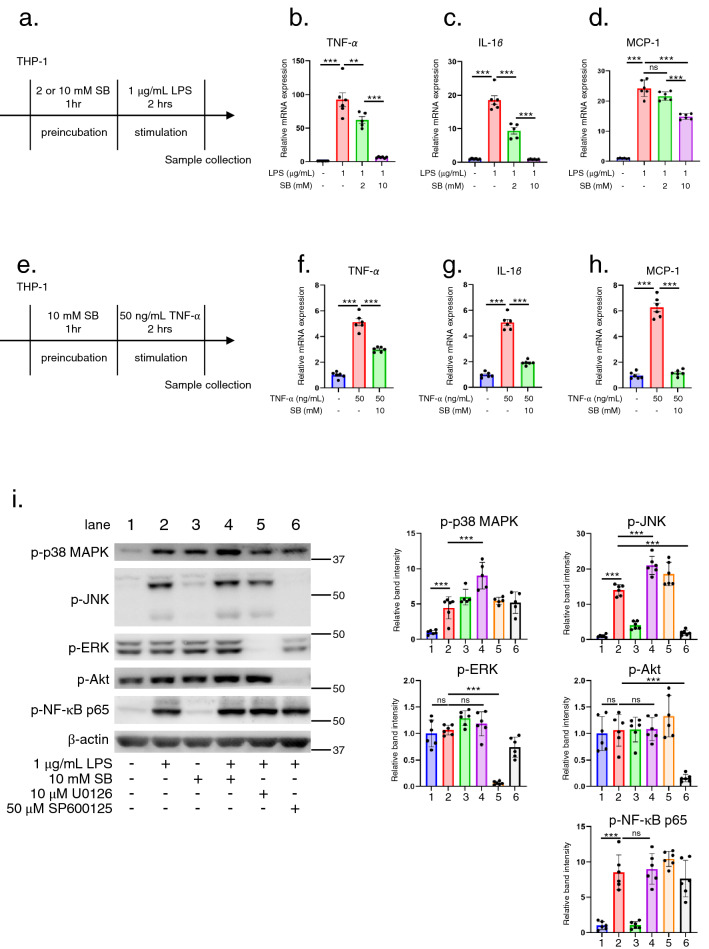
SB reduced the expression of inflammatory genes in stimulated THP-1 cells. (**a**) Protocol for LPS stimulation in THP-1 cells with or without SB preincubation. RT-PCR analysis of (**b**) *TNF-α*, (**c**) *IL-1β*, and (**d**) *MCP-1*. mRNA levels were adjusted to that of *GAPDH* (n = 5–10 each). (**e**) Protocol for TNF-α stimulation in THP-1 cells with or without SB preincubation. RT-PCR analysis of (**f**) *TNF-α*, (**g**) *IL-1β*, and (**h**) *MCP-1.* mRNA levels were adjusted to that of *GAPDH* (n = 6 each). (**i**) Immunoblotting (left panel) and quantification (right panel) of phosphorylated signal transduction molecules (n = 5–6). Band intensities were quantified using ImageJ software and adjusted to the intensity of the internal control molecule, β-actin. LPS (lane 2) significantly increased phospho-JNK and phospho-NF-κB p65 compared with the control (lane 1). SB did not decrease the phosphorylation of either molecule (lane 4). For comparison, the MEK inhibitor U0126 significantly decreased phospho-ERK levels (lane 5), while the JNK inhibitor SP600125 significantly decreased phospho-JNK (lane 6) and phospho-Akt (lane 6) levels. *p < 0.05; **p < 0.01; ***p < 0.001; ns, not significant. Each bar represents mean ± SEM. LPS, lipopolysaccharide; SB, sodium benzoate.

To investigate the direct effects of SB on the expression of inflammatory genes, we stimulated the human monocytic cell line, THP-1, with LPS (Fig. 5a) or TNF-α (Fig. 5e), because their respective receptors, TLR4^7^ and TNFR1^9^, have been implicated in the pathogenesis of adenine-induced nephropathy. LPS increased the mRNA levels of inflammatory genes such as *TNF-α* and *IL-1β*, as well as that of chemokine *MCP-1*, with the levels being significantly decreased by SB (Fig. 5b–d). TNF-α induced the expression of *TNF-α*, *IL-1β*, and *MCP-1* significantly, but this was significantly inhibited by SB (Fig. 5f–h). These data suggest that SB inhibits the expression of inflammatory genes in monocytic human THP-1 cells.

To closely examine the SB inhibitory mechanism, THP-1 cells were stimulated with LPS for 15 min and the phosphorylation levels of MAPK, Akt, and NF-κB p65 were measured (Fig. 5i). Phospho-ERK and phospho-Akt were abundantly expressed in baseline THP-1 cells, consistent with previous immunoblot experiments^27^. LPS upregulated phospho-JNK and phospho-NF-κB p65 levels compared with those in the control (lane 1 vs. 2). SB treatment did not decrease phosphorylation (lane 4), whereas the MEK inhibitor U0126 (lane 5) and JNK inhibitor SP600125 (lane 6) significantly inhibited phospho-ERK and phospho-JNK expression, respectively. These results are discrepant to those obtained in AdCKD mice experiments where both MAPK and NF-κB p65 signals are inhibited (Fig. 3). Since MAPK and NF-κB p65 signals are not only required for proinflammatory gene transcription but also downstream signaling elicited by these genes^25,26^, SB may inhibit primarily the expressions of proinflammatory cytokine expression in AdCKD mice thereafter block downstream signaling of these cytokines.

### SB reduces THP-1 cell motility

**Figure 6:**
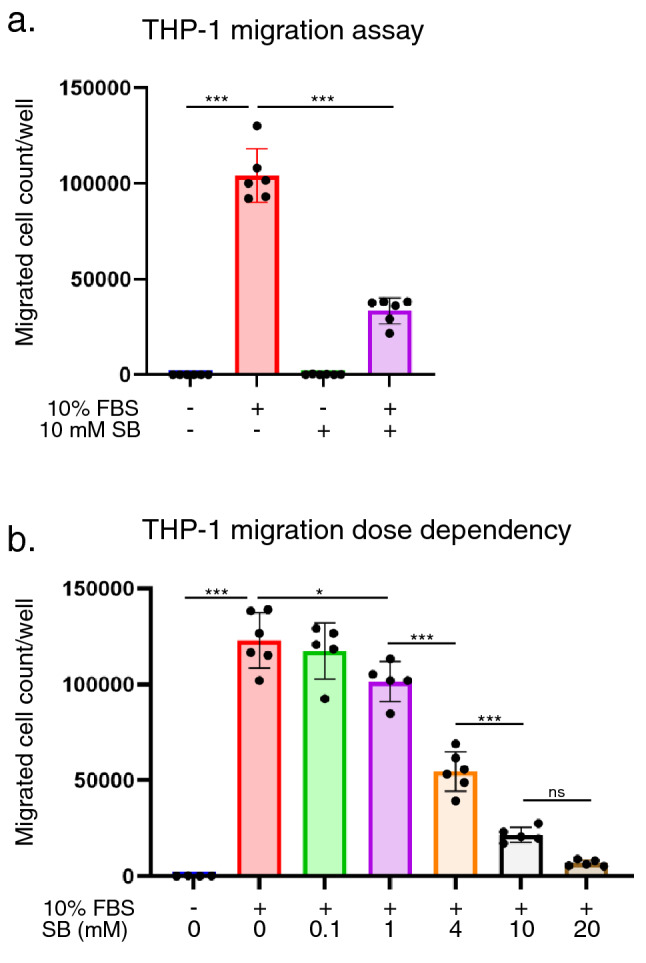
Transwell assay shows that SB reduces THP-1 migration. (**a**) The total number of cells in each well in the lower chamber of the 5 μm-pore transwell were counted and plotted after 24 h of incubation (n = 6 each). Cell counts per well were significantly lower in the FBS + SB group compared with the FBS group. (**b**) SB dose dependency was confirmed by applying different concentrations of SB to the upper chamber of the transwell (n = 4–6). Higher SB concentrations significantly reduced the number of migrating cells. *p < 0.05; ***p < 0.001; ns, not significant. Each bar represents mean ± SEM. FBS, fetal bovine serum; SB, sodium benzoate.

Since the ability of monocytes to mobilize and traffic themselves is one of the central functions driving inflammatory diseases^28^, we confirmed the inhibitory effect of SB on THP-1 cell motility using Transwell assays. When the cells were incubated with SB, the increase in THP-1 cell migration induced by FBS was significantly attenuated in a dose-dependent manner (Fig. 6a,b), confirming the inhibitory effect of SB on cell motility. These results were consistent with in vivo data that showed that the expression of *MCP-1* and *ICAM-1*, which are crucial adhesion molecules in monocytes^29,30^, was downregulated by SB treatment of AdCKD mice (Fig. 3n).

### SB reduces NF-κB RelB level of protein, mRNA, and binding to motif DNA sequence

**Figure 7:**
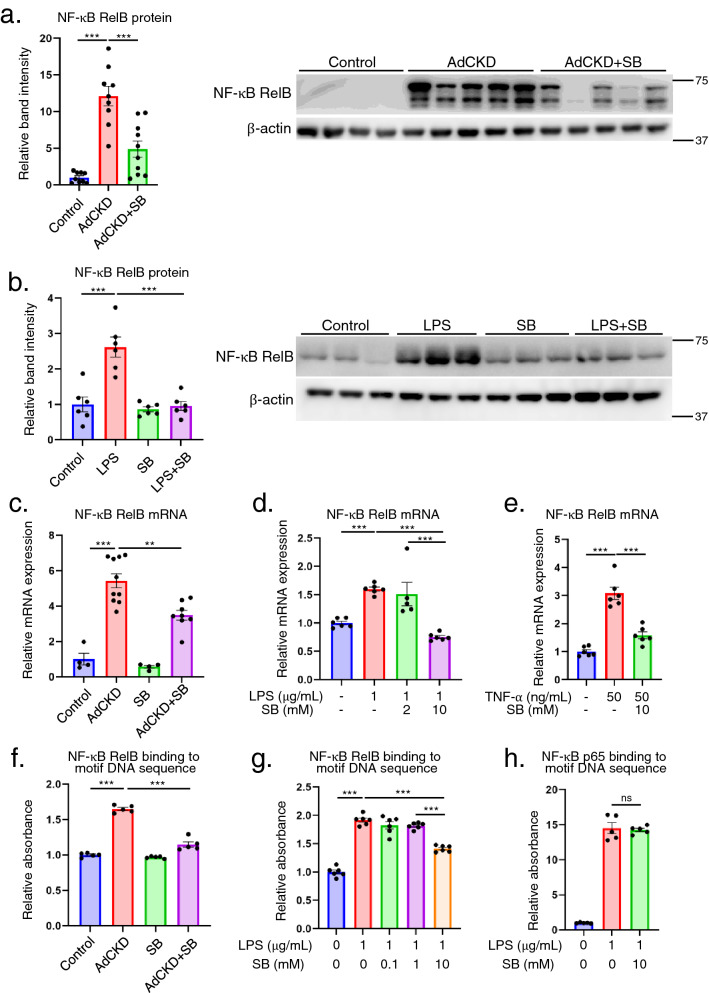
SB reduces NF-κB RelB levels of protein, mRNA, and binding to motif DNA sequence. (**a**) Band intensities of NF-κB RelB relative to beta-actin were plotted. In AdCKD kidney, NF-κB RelB protein expression was increased compared to control kidney, which was significantly decreased in AdCKD + SB kidney. (**b**) NF-κB RelB protein expression was increased in LPS stimulated THP-1 cells compared to control cells, which was significantly decreased in SB treated LPS stimulated THP-1 cells. (**c**) mRNA levels of NF-κB RelB in AdCKD kidney were increased compared to control, which was significantly decreased in AdCKD + SB. (**d**) mRNA levels of NF-κB RelB in LPS stimulated THP-1 cells were increased compared to control, which was significantly decreased in SB-treated LPS stimulated cells in a dose dependent manner. (**e**) mRNA levels of NF-κB RelB in TNF-α stimulated THP-1 cells were increased compared to control, which was significantly decreased in SB-treated TNF-α stimulated cells. (**f**) NF-κB RelB binding to motif DNA sequence was increased in AdCKD kidney compared to control, which was significantly decreased in AdCKD + SB kidney. (**g**) NF-κB RelB binding to motif DNA sequence was increased in LPS stimulated THP-1 cells compared to control, which was significantly decreased in SB-treated LPS stimulated cells in a dose dependent manner. (**h**) NF-κB p65 binding to motif DNA sequence was increased in LPS stimulated THP-1 cells compared to control, which was similar levels compared to SB-treated LPS stimulated cells. **p < 0.01; ***p < 0.001; ns, not significant. Each bar represents mean ± SEM. LPS, lipopolysaccharide; TNF-α, tumor necrosis factor α; SB, sodium benzoate.

To investigate the underlying mechanism for SB reducing the inflammatory gene expression, we focused on NF-κB RelB, as an upstream molecule associated with inflammatory gene regulation^31,32^. Protein level of NF-κB RelB was increased significantly in AdCKD kidneys compared to controls, which was significantly decreased in AdCKD + SB compared to AdCKD (Fig. 7a). Protein level of NF-κB RelB showed similar results in LPS stimulated THP-1 cells (Fig. 7b). mRNA levels of NF-κB RelB also showed similar results both in vivo and in vitro (Fig. 7c–e). We also confirmed that the mRNA level of NF-κB RelB was decreased in THP-1 cells treated with TNF-α + SB compared to TNF-α alone (Fig. 7e). The binding capacity of NF-κB RelB to its motif DNA sequence was increased in AdCKD kidneys compared to controls whereas it was significantly decreased in AdCKD + SB (Fig. 7f). The binding also was decreased in LPS stimulated THP-1 cells (Fig. 7g). We also confirmed that SB inhibition of motif DNA sequence binding was specific to NF-κB RelB because NF-κB p65, which is also an upstream inflammatory regulatory molecule, was unaltered by SB treatment in LPS stimulated THP-1 cells (Fig. 7h). Figure 7d and g presents that the inhibiting effect of NF-κB RelB by SB showed dose dependency.

## Discussion

**Figure 8:**
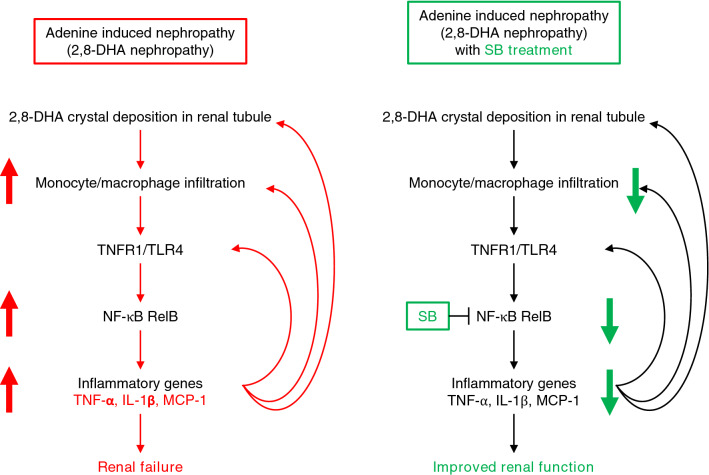
Graphical abstract showing the beneficial effects of SB in the kidneys of AdCKD mice. Left panel: In AdCKD mice, 2,8-DHA crystal deposits accumulated in renal tubules leads to monocyte/macrophage infiltration into the kidney tissue following upregulated expression of inflammatory genes such as TNF-α, IL-1β, and MCP-1, as well as TNFR1 and TLR4. In this process, NF-κB RelB is also increased. The upregulated inflammatory genes positively regulate additional crystal deposition^7^, monocyte/macrophage infiltration, and the expression of inflammatory genes. This positive feedback loop resulted in renal failure. Right Panel: SB treatment reduces the expression of NF-κB RelB and inflammatory genes, thereby down regulating monocyte/macrophage infiltration and the expression of the inflammatory genes, improving renal function. AdCKD, adenine induced chronic kidney disease; SB, sodium benzoate; CKD, chronic kidney disease; TNFR1, tumor necrosis factor receptor 1; TNF-α, tumor necrosis factor α; IL-1β, Interleukin 1 beta; MCP-1, Monocyte chemoattractant protein-1; TLR4, Toll-like receptor 4.

We have presented data showing that AdCKD can be improved by orally administering SB to mice, independent of their DAO enzymatic activity. *TNF-α* (Fig. 3g), *IL-1β* (Fig. 3h), and *MCP-1* (Fig. 3i) expression, and MAPK (Fig. 4c,d) and NF-κB p65 (Fig. 4f) phosphorylation in the kidneys of SB-treated AdCKD mice were significantly downregulated. As a result, macrophage infiltration and kidney fibrosis were attenuated (Fig. 3a), resulting in improved kidney function (Fig. 1c,d). Studies have shown that when the expression of various inflammatory and chemotactic genes is induced and inflammation is amplified once the input signal crosses a given threshold^33,34^, forming a positive feedback loop. In the kidneys of AdCKD mice, the expression of these inflammatory genes influenced the infiltrated monocytic cells to develop into M1 macrophages, which released cytokines that inhibit the proliferation of surrounding cells and damaged contiguous tissue^35^. M2 macrophages released cytokines that promote tissue repair in response to M1 macrophages^35^. These cytokines include TGF-β, a critical regulator of kidney fibrosis^36^. Our study showed that both the M1 macrophage marker CD80 (Fig. 3d) and the M2 macrophage markers CD163 and CD206 (Fig. 3e and f, respectively) were upregulated in AdCKD mice, suggesting that both the inflammatory and repair processes caused by these macrophages are activated. TNF-α signaling directly contributes to the development of inflammation in the kidney of AdCKD by increasing the crystal deposition area^7^, whereas IL-1β signaling promotes kidney fibrosis^37^, both of which were significantly suppressed by SB. Additionally, MCP-1, which upregulates TNF-α, IL-1β, and TGF-β^38^, was suppressed by SB. MCP-1 is associated with inflammation and CKD progression in various human and experimental kidney diseases^39,40^. The suppression of these pro-inflammatory molecules by SB contributed to the blockade of the positive feedback loop of inflammation in AdCKD mice, preventing the progression of kidney injury and renal failure (Fig. 8).

SB reduced the expression of inflammatory molecules such as iNOS and IL-1β, and increased the expression of contact molecules such as ICAM-1 and E-selectin in a mouse model of experimental allergic encephalomyelitis^17^. SB also reduced TNF-α levels following LPS stimulation in mouse BV-2 microglial cells^18^. In this study, we determined that the inhibitory effect of SB on AdCKD was due to the suppression of activated monocytic cells (Fig. 5). The expression of inflammatory genes, including *TNF-α*, was upregulated by LPS or TNF-α stimulation in THP-1 cells, which was significantly downregulated by SB treatment (Figure 5b–d, f–h). *TNF-α* was inhibited under every experimental condition, thus leading to significantly improved AdCKD in SB treated mice compared to AdCKD mice because TNF-α plays a central role in adenine-induced nephropathy pathophysiology as reported previously^7^.

A mechanism for SB inhibition of inflammation has been proposed. Based on previous reports^18,41^, benzoate is converted into benzoyl-CoA, which inhibits the mevalonate pathway, thereby inhibiting phenyl pyrophosphate production, resulting in inhibition of the Ras-ERK/MAPK cascade and downstream NF-κB-targeted gene transcription. However, these studies focused on the NF-κB reporter or iNOS expression and did not measure the phosphorylation of ERK or NF-κB. Our in vivo analysis showed that phosphorylation of ERK, NF-κB p65, and JNK was attenuated in the kidneys of SB-treated AdCKD mice (Fig. 4). In contrast, in vitro analysis of THP-1 cells showed that SB treatment did not inhibit either MAPK or NF-κB p65 phosphorylation (Fig. 5i). Generally, inflammatory molecules such as TNF-α, IL-1β, MCP-1, and TLR4 have downstream mediators such as MAPK and NF-κB signal^25,26^, and activation of MAPK and NF-κB signals are, in turn, necessary to increase the mRNA transcription of inflammatory molecules such as TNF-α, IL-1β, and MCP-1^25,26^. Taken together, the inhibition of ERK, JNK, and NF-κB p65 phosphorylation in SB-treated AdCKD mice can be interpreted as a secondary inhibitory effect of SB on the mRNA expression of *TNF-α, IL-1β*, and *MCP-1* rather than direct effect. Finally, we have successfully demonstrated that SB inhibits NF-κB RelB, which is an upstream regulatory molecule for broad inflammatory molecules including TNF-α, IL-1β, and MCP-1^31,32^. Taken all together, SB inhibition of proinflammatory cytokines is due to inhibited NF-κB RelB expression, ultimately leading to alleviated kidney injury in AdCKD mice model.

In addition to its anti-inflammatory action, SB inhibited the motility of THP-1 monocytic cells (Fig. 6). Cell migration and trafficking are multistep processes that involve local recruitment of inflammatory cells^28^. Recruited monocytes can participate in the initial inflammatory response by releasing TNF-α and IL-1β, as well as pattern recognition receptors such as TLRs^42^. MCP-1 is a chemoattractant that induces THP-1 cell migration^43^. Thus, cell motility is significant in initiating inflammation. AdCKD pathophysiology is associated with inflammatory molecules such as TNF-α^7^ and NF-κB^9^ and chemotactic molecules such as CCL3 and CCR5^14^. The beneficial effect of SB in AdCKD seems to be associated with a reduction in the chemotactic molecule, MCP-1 (Fig. 3i) and the trafficking molecule, ICAM-1 (Fig. 3n). Additionally, in vitro analysis showed that SB inhibited the transcription of inflammatory genes which was activated upon LPS or TNF-α stimulation (Fig. 5). SB also inhibited THP-1 cell motility (Fig. 6). Since cell motility and inflammation are interrelated^28,42^, the presented results show that SB inhibited mRNA of several proinflammatory molecules through inhibition of NF-κB RelB, leading to down regulated cell motility.

The potential limitation of this study is that, first, SB could exhibit hematological abnormality including anemia in mice^44^, therefore SB usage should be handled with caution to avoid overdose. However, the average dosage of SB in our study was 500 mg/kg/day per mouse which human equivalent dose is 40 mg/kg/day, and this is only 16 percent of the admitted human dose of SB in human urea cycle disorder of 250 mg/kg/day^45^. The side effect may not be significant, although, long-term administration side effects should carefully be determined if applying to diseases including AdCKD and human CKD related to APRT deficiency in the future. Secondly, we have not presented the blood concentration of SB in mice, so this could be a limitation in this study. Besides, as presented in Fig. 1j, we have confirmed the dose dependency of SB on kidney function. This could support that blood concentration of SB is adequately elevated triggering beneficial effects to kidney function in AdCKD. Also, according to Fig. 1j, the minimal effective dose of SB in AdCKD could be considered as 5 mM, which triggered reduction of both plasma urea nitrogen and creatinine. Thirdly, since this study focused on treating AdCKD mouse model, we have not conducted experiments for human participants. Future study should aim to treat human CKD patients. Lastly, we have employed modified experimental protocol for obtaining MCP-1 mRNA level in THP-1 cells stimulated by LPS as shown in the method section, in which we used medium without FBS. Using the FBS containing medium during LPS stimulation made MCP-1 increase less detectable compared to control^46^, because FBS itself induces MCP-1^47^. This is not the case for TNF-α or IL-1β, because FBS reduces the level of these molecules^48,49^. The modified protocol successfully increased the level of MCP-1 mRNA by LPS, which was significantly reduced by SB (Fig. 5d).

This study is novel because DAO-deficient mice were used to confirm the therapeutic effect of SB on AdCKD. Although SB is a well-known DAO inhibitor^16^, previous studies have not examined the relevance of DAO. We have clearly shown that the beneficial effect of SB on AdCKD is independent of DAO. Additionally, we have presented that the underlying mechanism of inhibiting inflammation by SB involves inhibited NF-κB RelB, an upstream inflammation regulatory molecule. SB exerts beneficial effects by reducing inflammation and protecting the kidneys during diseases such as AdCKD and opens future investigations in human CKD related to APRT deficiency.

## Materials and methods

### Animal experiments

All animal experiment protocols were approved by the Institutional Animal Care and Use Committee of Keio University (Tokyo, Japan) (Approval No. 21011-(0)), and all experiments were performed following relevant guidelines and regulations and in compliance with the ARRIVE guidelines. C57BL/6JJcl-specific pathogen-free (SPF) male mice (CLEA Japan, Tokyo, Japan) were fed a standard CE-2 diet (CLEA Japan) and provided ad libitum access to tap water. Seven-week-old mice were randomly assigned into four groups with or without adenine in chow and with or without sodium benzoate (SB) (20 mM) in drinking water: control group, 2,8-DHA nephropathy group (AdCKD group), SB-treated control group (SB group), and AdCKD with SB treatment group (AdCKD + SB group) (Fig. 1a). Each group consisted of ten mice each. Five mice were kept in each cage. Mice cages were made of transparent plastic supplemented with chip bedding and the cages were randomly placed in a rack to minimize potential confounders such as location of a cage. Mice weighted between 18 and 24 g were used. Mice in the control group were fed a normal diet whereas those in the AdCKD group were given CE-2 supplemented with 0.2% adenine (Wako, Japan) for six or eight weeks. SB was dissolved in distilled water to give final concentrations of 0.1 mM, 1 mM, 5 mM, and 20 mM. These were used for dose-dependent analysis, and each group consisted of four to five mice each. Survival analysis was performed using ten mice from each group who were monitored over a 336-day period. Mice were monitored if they were alive every day and the date of the demise of a mouse was recorded as the endpoint of the analysis. Demise of a mouse was defined as confirmation of postmortem rigidity or whole-body tremor which was considered a humane endpoint. Finally, the mice were weighed, euthanized using intraperitoneal injection of 0.3 mg/kg medetomidine, 4.0 mg/kg midazolam, and 5.0 mg/kg butorphanol, and all efforts were made to minimize animal suffering. Blood samples were collected from the inferior vena cava and sacrificed. Kidney tissues were extracted and snap frozen in liquid nitrogen and stored at − 80 °C for further use. Serum creatinine and urea nitrogen (UN), urine creatinine, total protein, and N-acetyl-β-D-glucosaminidase (NAG) were measured as described previously^50^. Kidneys which developed hydronephrosis were not included in the study. Blinding was not conducted in the experiments. No adverse events were observed.

### DAO mouse line carrying a G181R mutation

A ddY/DAO − mouse line lacking DAO activity due to a G181R point-mutation in the DAO gene (DAO − / − ) was backcrossed with C57BL/6JJcl mice 15 times, as described previously^21^. The female mice were crossed with C57BL/6JJcl male mice to obtain a heterozygous DAO + / −  mouse strain. The G181R mutation was genotyped in littermates of DAO + / − crosses. The littermates were then divided into wild-type (DAO + / +), heterozygous DAO G181R (DAO + / − ), or homozygous DAO G181R (DAO-/-) groups (n = 6–17 per group). The mice were fed with CE-2 chow containing 0.2% adenine to induce AdCKD. DAO − / − mice were divided into three groups (n = 4–11 per group): control, 0.2% adenine-induced CKD (AdCKD group), and 0.2% adenine-induced CKD treated with 20 mM sodium benzoate (AdCKD + SB group). Blood was collected similarly. For genotyping, the tails of the mice were cut and vortexed in 50 mM NaOH (180 μL) and then incubated at 95 °C for 10 min. The reaction was stopped by adding 1 M Tris–HCl (pH 8.0, 20 μL). Two microliters of the sample lysate were used as a PCR template, which was performed using Quick Taq® HS DyeMix DTM-101 (Toyobo, Japan). Nested PCR was performed using the following primers: 1st PCR forward 5ʹ-GAAGAGGGAGAGGAGGAGAAT-3ʹ and reverse 5ʹ-TTTGGTTAAGATGGTGATGTG-3ʹ; 2nd PCR forward 5ʹ-GGGAGAGGG CACAGCACAGTC-3ʹ, reverse 5ʹ-ACACCAGGGCAGGAGTAGGC-3ʹ. PCR products were electrophoresed on a 3% agarose gel containing 0.1% ethylene bromide. Bands were detected under UV light. The product of the 1^st^ PCR was diluted 200 times and used as a template in the 2nd PCR.

### Histological staining and assessment

The kidney tissues were fixed in 10% formalin neutral buffer solution (Fujifilm, Japan) and embedded in paraffin. 4-μm slices were stained with masson trichrome (MT) according to a standard protocol. For F4/80 immunohistochemistry (IHC) staining, 4-μm slices of the paraffin-embedded tissues were processed as follows: deparaffinized slices were treated with proteinase K and 3% hydroxy peroxide for antigen retrieval. The slices were incubated in anti-mouse F4/80 rat monoclonal antibody (Bio-Rad, 1:200) at 20 to 25 °C for 50 min, washed in PBS, enhanced by histofine simple stain max-po (Nichirei Bioscience, Japan), and stained with 3,3'-diaminobenzidine and then with Mayer's hematoxylin nucleus stain. Digital images were obtained using a NanoZoomer-XR C12000 virtual slide scanner (Hamamatsu Photonics K.K., Japan). Ten high power field pictures of every MT slide were captured for the assessment. Each picture was given an interstitial fibrosis and tubular atrophy (IFTA) score based on a previously described criteria^51^. Briefly, IFTA scores were assigned as follows: 0 for images with < 25% renal tissue affected, 1 for images with 25–50% of the renal tissue affected, 2 for images with 50–75% of the renal tissue affected, and 3 for images with 75% or more of the renal tissue affected. Five images of each stained kidney were captured for IHC analysis. Color deconvolution was applied to each image using ImageJ software (National Institutes of Health, USA) using a color deconvolution plug, after the 3,3″-diaminobenzidine tetrahydrochloride area was calculated. Antibody details are provided in Table S2.

### Cell culturing and stimulation

Cells from the human monocytic leukemia cell line THP-1 were grown in RPMI-1640 medium (ATCC, USA) supplemented with 10% fetal bovine serum (FBS) at 37 °C in the presence of 5% CO_2_. THP-1 cells (1 × 10^6^ cells/mL) were stimulated with 1 µg/mL LPS or 50 ng/mL TNF-α for 2 h. For Fig. 5d, THP-1 cells were incubated in medium without FBS for 10 h and stimulated with 1 µg/mL LPS for 6 h. SB was pre-treated for 1 h before cell stimulation. RNA was subsequently extracted from the cells. For immunoblotting, THP-1 cells were stimulated with 1 µg/mL LPS for 15 min. For Fig. 7, THP-1 cells were stimulated with 1 µg/mL LPS for 2 h.

### THP-1 cell migration assay

Migration assays were performed in a 5-µm diameter Transwell (Corning, Corning, NY, USA). THP-1 monocytes (2 × 10^5^ cells) were suspended in serum-free RPMI-1640 medium (200 µL) in the upper chamber before and after preincubating with 10 mM SB for 1 h. RPMI-1640 medium (600 µL) with or without 10% FBS was added to the lower transwell chamber. The medium in the lower chamber was collected and centrifuged (4 °C, 5000 *g*, 5 min) after 24 h. The supernatant was removed, and 0.4% trypan blue stain (Gibco, USA) used to stain the cells. The total number of cells was counted using a hemocytometer and the total cell count per well was plotted.

### RNA extraction and real-time polymerase chain reaction (RT-PCR)

RNA extraction and quantitative real-time PCR were performed as follows: briefly, RNA was purified from mouse kidney homogenates using the RNeasy Mini Kit (Qiagen, Germantown, MD, USA) and from cell lysates using TRIZOL (Invitrogen, Waltham, MA, USA). mRNA concentration was measured using a Nanodrop One C (Thermo Fisher Scientific, Waltham, MA, USA). RNA (500 μg) was reverse transcribed into cDNA using PrimeScript RT Master Mix (Takara, Japan). PCR was performed on a StepOnePlus Real-Time PCR system (Applied Biosystems, Waltham, MA, USA) using PowerUp SYBR Green Master Mix (Thermo Fisher Scientific). mRNA levels were normalized to those of the housekeeping gene *HPRT* (mouse kidney assays) or *GAPDH* (in vitro analysis), and the levels relative to those in the control group were plotted. Expression levels were calculated using the ΔΔCT method. The sequences of the primers used are listed in Table S1.

### Immunoblotting

Immunoblotting was performed as follows: briefly, kidney lysate was obtained by homogenizing kidney cortex tissue in modified RIPA buffer (50 mM Tris–HCl [pH 8.0], 150 mM NaCl, 1% NP-40, 5 mM EDTA, 5 mM MgCl_2_) supplemented with protease inhibitor cocktail (Roche, Basel, Switzerland), 20 mM NaF, 0.5 mM PMSF, 10 mM nicotinamide, and 330 nM Trichostatin A. Homogenates were centrifuged at 4 °C and 12,000 *g* for 15 min. Protein concentration in the supernatant was measured using the Bradford method. Sample supernatants were incubated at 95 °C for 5 min with Laemmli sample buffer (Bio-Rad, Hercules, CA, USA) mixed with 2-mercaptoethanol to denature proteins. Protein extracts were separated via electrophoresis on 8–15% sodium dodecyl sulfate–polyacrylamide gels. Proteins were transferred from the gels to PVDF membranes (Bio-Rad) using a transblot turbo system (Bio-Rad). The membranes were blocked with 5% skim milk or EzBlock Chemi (Atto, Japan) for 1 h at 20–25 °C and then incubated overnight at 4 °C with primary antibodies diluted (1:1000) with either 5% skim milk or Can Get Signal Immunoreaction Enhancer Solution (Toyobo, Japan). The membranes were thereafter washed three times in TBST (0.1% tween-20 in TBS), incubated for 1 h at room temperature with diluted (1:5000) secondary antibody, and then washed three times with TBST. Chemiluminescence was detected using ECL prime (Cytiva, Malborough, MA, USA) and imaged using the LAS-4000 mini (GE healthcare, Chicago, IL, USA). Band intensities were quantified using ImageJ software and normalized to those of internal proteins. Antibody specifications are listed in Table S2.

### Immunofluorescent of kidney tissue, nitrite production, DAO activity assay, and assay of NF-κB binding to motif DNA sequence

These protocols are described in the Supplementary Methods.

### Statistical analysis

Data are expressed as mean ± SEM and analyzed by the student’s t test for two group comparison and by the Tukey–Kramer test for multiple group comparisons. Survival was analyzed using log-rank test. The sample size was determined empirically and confirmed by samplesize calculator (http://powerandsamplesize.com/) using power of 0.8, α of 0.05, standard deviation of 0.1 to 1. Statistical significance was set at *p* < 0.05. All analyses were performed, and graphs were generated using either EZR version 1.35 (Saitama Medical Center, Jichi Medical University, Saitama, Japan), which is a graphical user interface for R (The R Foundation for Statistical Computing, Vienna, Austria) or GraphPad Prism, version 9 (https://www.graphpad.com/).

## Supplementary Information

Supplementary Information.

## Data Availability

The data that support the findings of this study are available from the corresponding authors upon request.
