# Supplementary material for: Sodium benzoate attenuates 2,8-dihydroxyadenine nephropathy by inhibiting monocyte/macrophage TNF-α expression
Source: Sci Rep. 2023 Feb 27;13:3331. doi: 10.1038/s41598-023-30056-6 (PMC9971245; doi:10.1038/s41598-023-30056-6)
Supplement: Supplementary file 1 — Supplementary Information. [file 41598_2023_30056_MOESM1_ESM.pdf]

Sodium benzoate attenuates 2,8-dihydroxyadenine nephropathy by inhibiting monocyte/macrophage TNF-α expression

Yoichi Oshima[1], Shu Wakino[2*], Takeshi Kanda[1], Takaya Tajima[1], Tomoaki Itoh[1], Kiyotaka Uchiyama[1], Keiko Yoshimoto[1], Jumpei Sasabe[3], Masato Yasui[3], Hiroshi Itoh[1]

[1]Department of Internal Medicine, Keio University School of Medicine, Tokyo, Japan

[2]Department of Nephrology, Tokushima University School of Medicine, Tokushima, Japan

[3]Department of Pharmacology, Keio University School of Medicine, Tokyo, Japan

Corresponding author: Shu Wakino, Department of Nephrology, Tokushima University School of Medicine, Tokushima, Japan

E-mail: shuwakino@tokushima-u.ac.jp

Supplementary Method

Kidney tissue immunofluorescent staining

Perfusion fixation was performed as follows. Briefly, mice were euthanized, and right atrium was incised, PBS were infused through a fine needle into the left ventricle to perfuse the entire blood vessels. After the blood was substituted by PBS, 4 % paraformaldehyde (Muto chemical, Japan) was perfused thoroughly. The kidney was excised, immersed with 4 % paraformaldehyde at 4 ℃ overnight, substituted with 20 % sucrose for 24 hours, then embedded in OCT compound (Sakura, Japan), slowly frozen with liquid nitrogen-chilled stainless tray, then stored at -80 ℃. 6 μm section was sliced by cryostat, dried, blocked by 3% BSA and 3% donkey serum in PBST (0.25% triton-X in PBS) for one hour at room temperature, reacted with primary antibody (1:100, anti-AQP-1, Proteintech) at 4℃ overnight, washed three time with PBST, reacted with secondary alexa-488 donkey anti-rabbit IgG antibody (Thermo Fisher Scientific) for one hour at room temperature, washed three times with PBST, then sealed by SlowFade® Diamond (Invitrogen). The immunofluorescent image was acquired by BZ-9000 (Keyence, Japan). Antibody specification is shown in Table 1.

Nitrite Production Assay

The nitric oxide (NO) levels in the kidney cortex were determined by the Griess reaction following manufacture protocol (Cayman Chemical, USA). Briefly, kidney cortex was homogenized in PBS and centrifuged to obtain supernatant and was mixed with Griess reagent in a 1.5 mL tube followed by incubation at 60 °C for one hour. The reaction solution was transferred to a 96-well plate. Subsequently the absorbance of 540 nm of the samples was determined. Protein concentration was measured by Direct Detect (Merck Millipore, USA). Nitric oxide content per protein unit was calculated and plotted.

DAO activity assay

Kidney homogenate DAO activity was measured by using D-Amino Acid Oxidase Activity Assay Kit (K559-100, Biovision, USA) according to manufacture protocol. Briefly, 10 mg of control mouse kidney was homogenized in 100 μL of ice cold DAAO assay buffer with dounce tissue homogenizer and kept on ice for 10 minutes followed by centrifugation (10000 g, 5 minutes). 1 μL of supernatant was added to each well. 2 μL of 1 mol/L SB was added for SB group. The reaction mix was applied for each well and immediately measured fluorescence (excitation wave 535 nm/ emission wave 587 nm) every 70 seconds for 30 minutes by plate reader. Initial linear portion of

production rate of hydrogen peroxide was calculated for each well. All experiment was duplicated, and the mean was plotted.

Assay of NF-κB binding to motif DNA sequence

Kidney or cell homogenate NF-κB binding to motif DNA sequence was measured by using TransAM NFκB Family (cat no. 43296, active motif, USA) according to manufacture protocol. Briefly, for kidney tissue, 3 to 5 mg of kidney tissue was homogenized in supplied lysis buffer. THP-1 cells were incubated with or without SB for 1 hour at 37 degrees followed by incubation with or without LPS for 2 hours, then collected by centrifugation at 5000 g, 5 minutes at 4 degrees, lysed by pipetting in supplied lysis buffer. Protein concentration was measured by bradford method or by using Direct Detect system (Merck Millipore, USA). 40 micrograms of protein were applied per well, incubated at 25 degrees Celsius for one hour, washed three times with supplied washing buffer, applied primary antibody at 25 degrees Celsius for one hour, washed three times with supplied washing buffer, applied secondary antibody at 25 degrees Celsius for one hour, three times with supplied washing buffer. Reacted with development solution for 5 minutes, then applied stop solution, followed by immediate measurement of absorbance at 450 nm.

Supplementary Table Legend

Table S1. List of primers used in this study. The DNA sequences of primers of the respective genes are listed.

Table S2. List of antibodies used in this study. Types of antibodies, sources, and identifiers are listed.

Table S1. Primers used in this study

Mouse Gene	Forward sequence	Reverse sequence
**CD206**	5′ -CCACTCTATCCACCTTCAC-3′	5′ -GCCTCAATCCAACCAAAC-3′
**CD80**	5′- CCCCAGAAGACCCTCCTGATAG-3′	5′ -CCGAAGGTAAGGCTGTTGTTTG-3′
**F4/80**	5′ -CCCAGCTTCTGCCACCTGCA-3′	5′ -GGAGCCATTCAAGACAAAGCC-3′
**HRPT**	5′ -TTGTTGTTGGATATGCCCTTGACTA-3′	5′ -AGGCAGATGGCCACAGGACTA-3′
**IBA-1**	5′- ATCAACAAGCAATTCCTCGATGA-3′	5′ -CAGCATTCGCTTCAAGGACATA-3′
**ICAM-1**	5′- AACTGTGGCACCGTGCAGTC-3′	5′ -AGGGTGAGGTCCTTGCCTACTTG-3′
**IL-10**	5′- GCCAGAGCCACATGCTCCTA-3′	5′ -GATAAGGCTTGGCAACCCAAGTAA-3′
**IL-1β**	5′- TCCAGGATGAGGACATGAGCAC-3′	5′ -GAACGTCACACACCAGCAGGTTA-3′
**IL-6**	5′- CAACCACGGCCTTCCCTACT-3′	5′ -TTCTCATTTCCACGATTTCCCA-3′
**Lcn2**	5′- GGCCTCAAGGACGACAACA-3′	5′ -TCACCACCCATTCAGTTGTCA-3′
**MCP-1/CCL2**	5′- CAGGTCCCTGTCATGCTTCT-3′	5′ -CCCATTCCTTCTTGGGGTCA-3′
**TGFβ1**	5′- TACGGCAGTGGCTGAACCAA-3′	5′ -CGGTTCATGTCATGGATGGTG-3′
**TNF-α**	5′- ACTCCAGGCGGTGCCTATGT-3′	5′ -GTGAGGGTCTGGGCCATAGAA-3′
**RelB**	5′- GGTACTGCTAGCCTTGTGGG-3′	5′- AGGTTGGCTTCGGAATGGAG-3′
**Human Gene**		
**GAPDH**	5′ -GCACCGTCAAGGCTGAGAAC-3′	5′ -TGGTGAAGACGCCAGTGGA-3′
**IL-1β**	5′ -CCAGGGACAGGATATGGAGCA-3′	5′ -TTCAACACGCAGGACAGGTACAG-3′
**MCP-1/CCL2**	5′ -CTGCTCATAGCAGCCACCTT-3′	5′ -CAGGTGACTGGGGCATTGAT-3′
**RelB**	5′- ATGGCATCGAGAGCAAAC-3′	5′- AGAGAAGAAGTCAGGGTCTG-3′
**TNF-α**	5′ -CCTCTCTCTAATCAGCCCTCTG -3′	5′ -GAGGACCTGGGAGTAGATGAG-3′

Table S2. List of antibodies used in this study

Antibodies	SOURCE	IDENTIFIER	
**Rabbit polyclonal anti-βactin**	Cell Signaling Technology	Cat# 4967, RRID:AB_330288	https://antibodyregistry.org/search.php?q=AB_330288
**Rabbit polyclonal anti-aquaporin-1**	Proteintech	Cat# 20333-1-AP, RRID:AB_10666159	https://antibodyregistry.org/search.php?q=AB_10666159
**Rabbit monoclonal anti-Phospho-p38 MAPK**	Cell Signaling Technology	Cat# 4631, RRID:AB_331765	https://antibodyregistry.org/search.php?q=AB_331765
**Rabbit monoclonal anti-Phospho-SAPK/JNK (Thr183/Tyr185)**	Cell Signaling Technology	Cat# 4668, RRID:AB_823588	https://antibodyregistry.org/search.php?q=AB_823588
**Rabbit monoclonal anti-Phospho-p44/42 MAPK (Erk1/2) (Thr202/Tyr204)**	Cell Signaling Technology	Cat# 4370, RRID:AB_2315112	https://antibodyregistry.org/search.php?q=AB_2315112
**Rabbit polyclonal anti-Phospho Akt (Ser473)**	Cell Signaling Technology	Cat# 9271, RRID:AB_329825	https://antibodyregistry.org/search.php?q=AB_329825
**Rabbit monoclonal anti-Phospho-NF-κB p65 (Ser536)**	Cell Signaling Technology	Cat# 3033, RRID:AB_331284	https://antibodyregistry.org/search.php?q=AB_331284
**Rat monoclonal anti-F4/80**	Bio-Rad	Cat# MCA497RT, RRID:AB_1102558	https://antibodyregistry.org/search.php?q=AB_1102558
**Rabbit monoclonal anti-NF-κB RelB**	Cell Signaling Technology	Cat# 10544, RRID:AB_2797727	https://antibodyregistry.org/search.php?q=AB_2797727
**Goat polyclonal anti-Rabbit IgG(H+L)**	Thermo Fisher Scientific	Cat# A-11034, RRID:AB_2576217	https://antibodyregistry.org/search.php?q=AB_2576217
**Goat polyclonal anti-Rabbit IgG(H&L)**	Abcam	Cat# ab6721, RRID:AB_955447	https://antibodyregistry.org/search.php?q=AB_955447

Supplementary Figure Legend

Figure S1 to S4. Immunoblotting picture of respective molecules (iNOS, GAPDH) which are shown in Figure 3l.

Table S5 to S13. Immunoblotting picture of respective molecules (phospho-p38 MAPK, phospho-JNK, phospho-ERK, phospho-Akt, phospho-NF-κB p65, βactin) which are shown in Figure 4a.

Table S14 to S22. Immunoblotting picture of respective molecules (phospho-p38 MAPK, phospho-JNK, phospho-ERK, phospho-Akt, phospho-NF-κB p65, βactin) which are shown in Figure 5i.

Table S23 and S24. Immunoblotting picture of NF-κB RelB and βactin which are shown in Figure 7a.

Table S25 and S26. Immunoblotting picture of NF-κB RelB and βactin which are shown in Figure 7b.

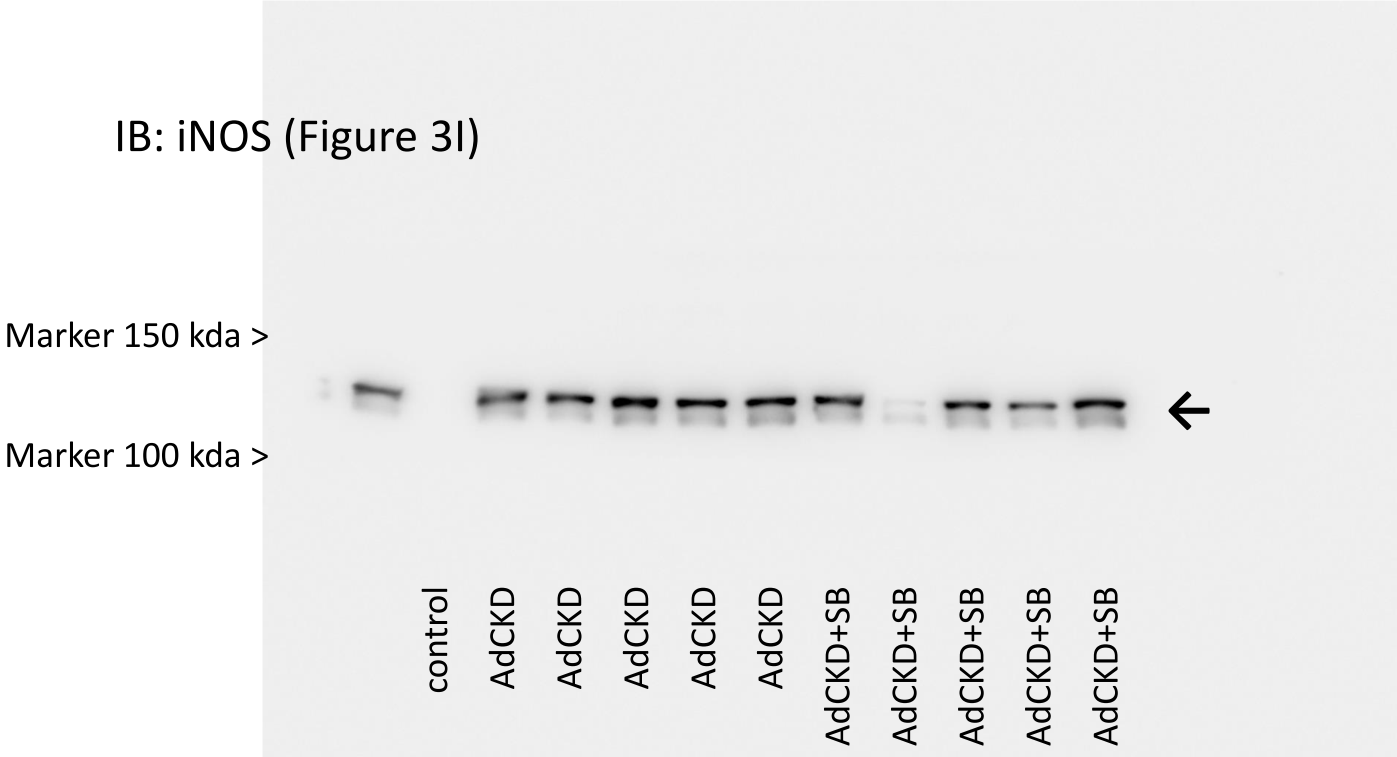

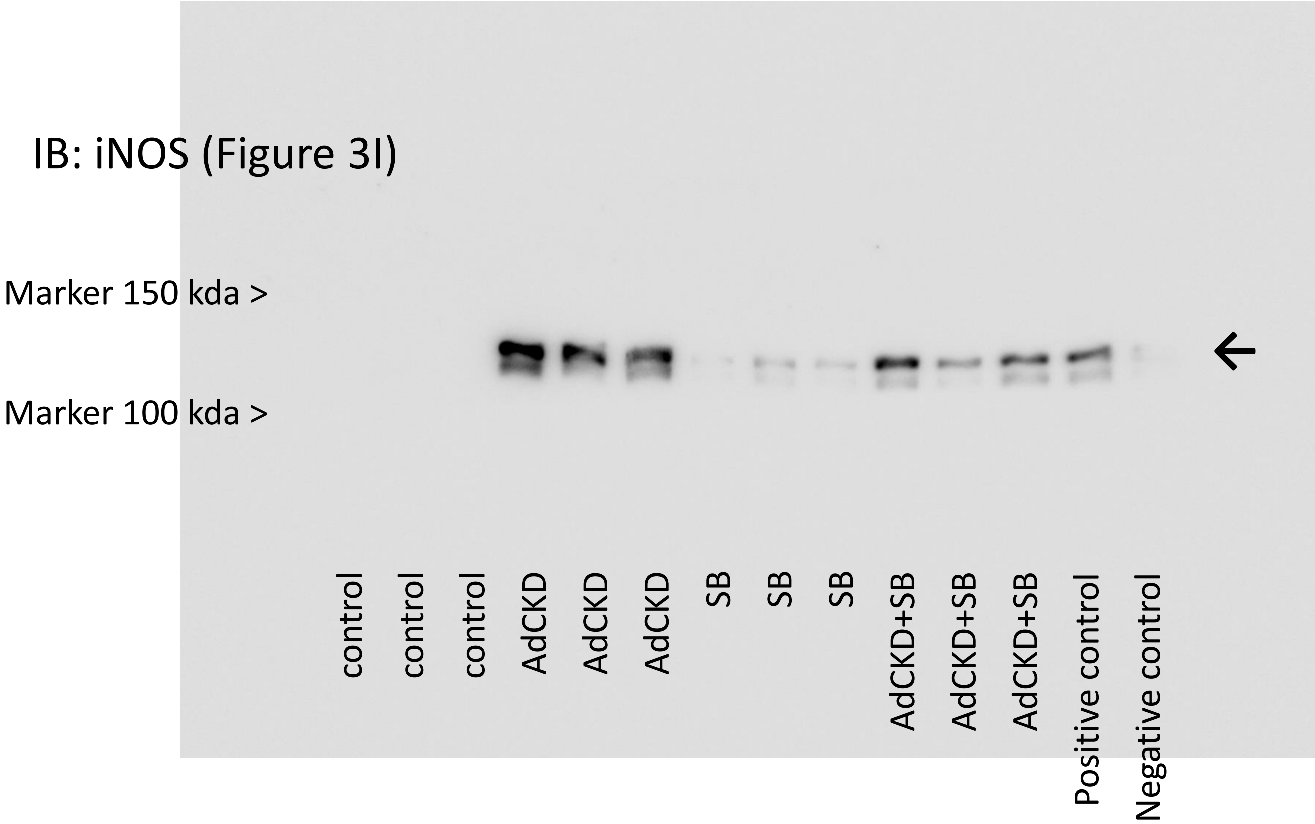

Supplementary Figure S1, Oshima, et al.

IB: iNOS (Figure 3I)

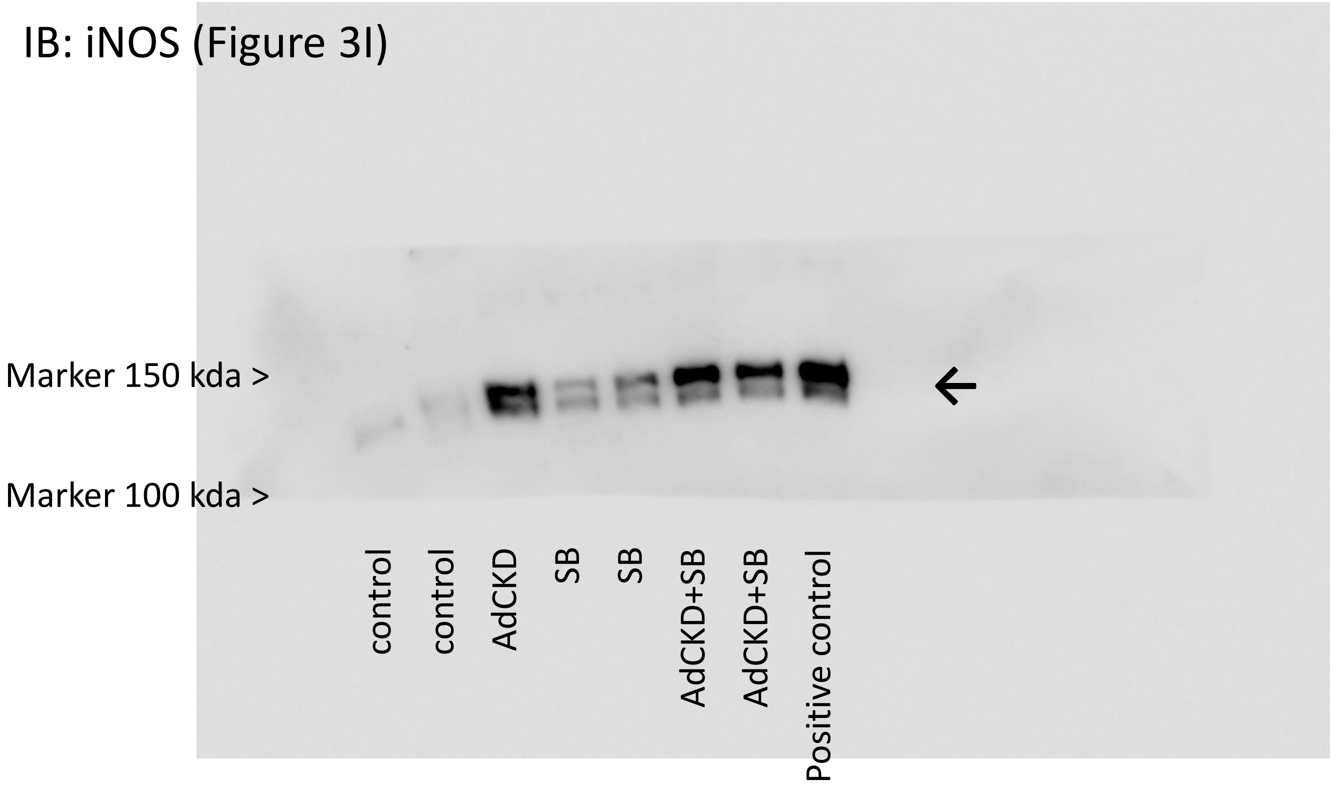

IB: iNOS (Figure 3I)

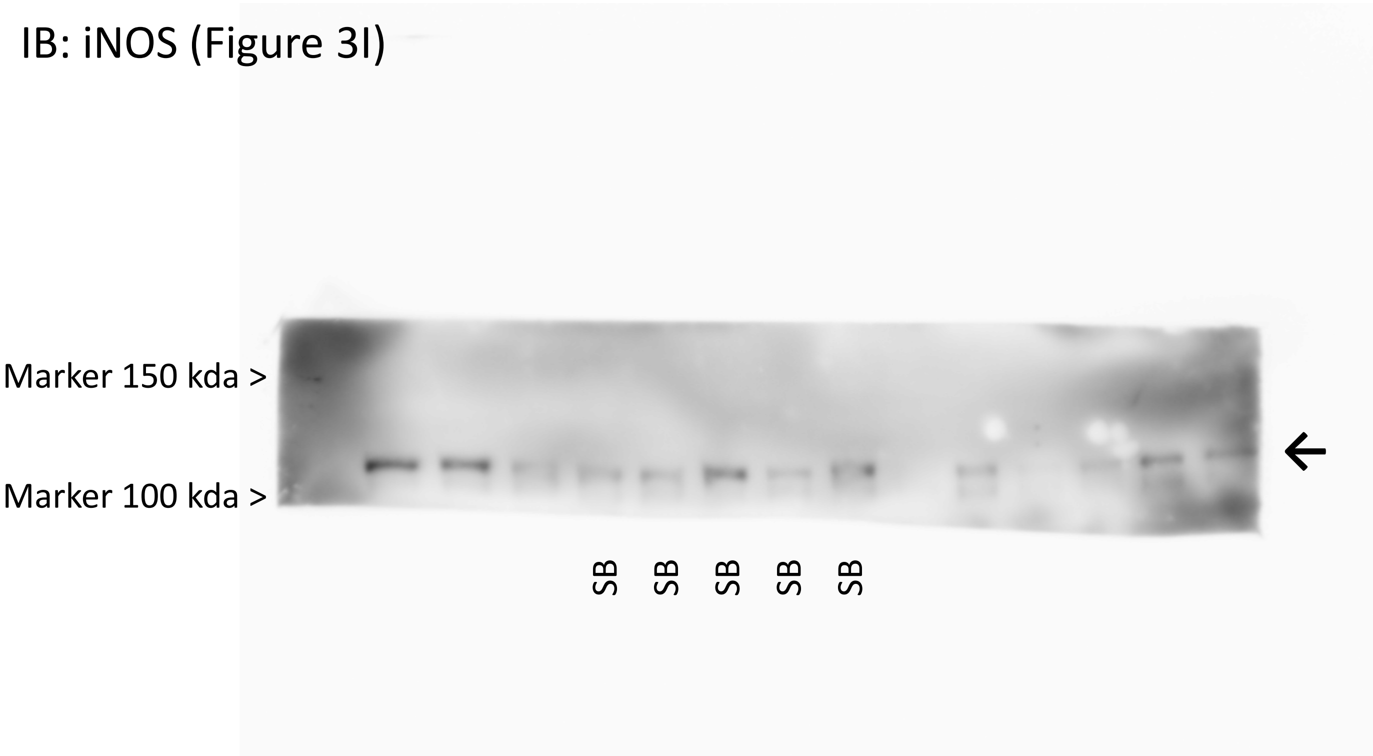

Supplementary Figure S2, Oshima, et al.

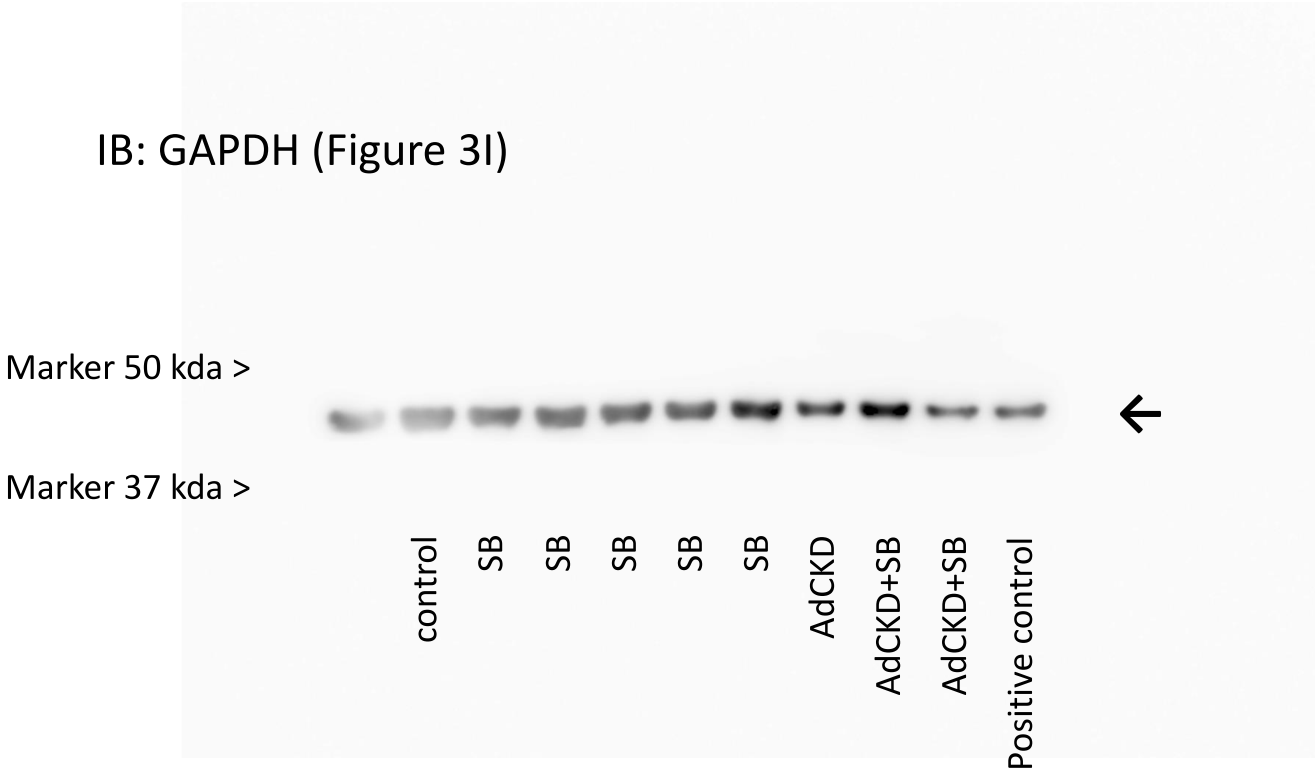

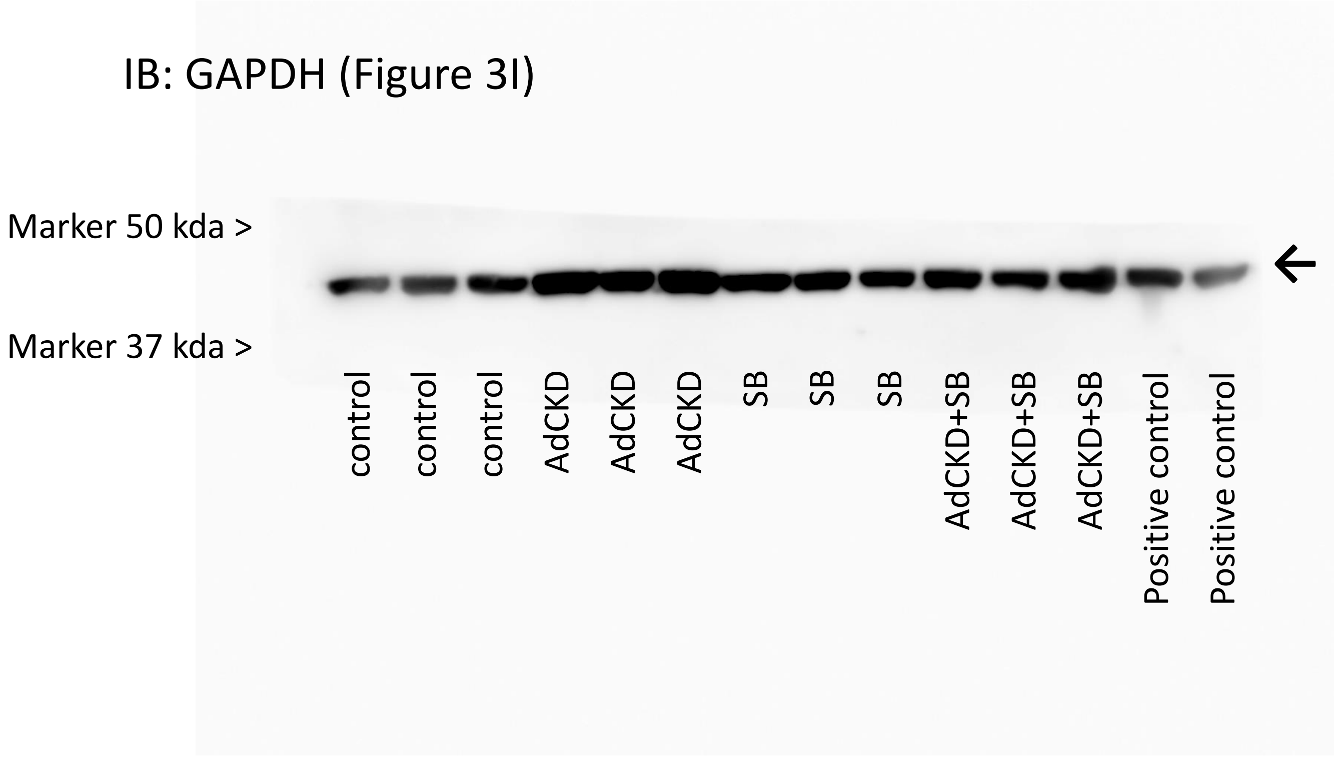

Supplementary Figure S3, Oshima, et al.

IB: GAPDH (Figure 3I)

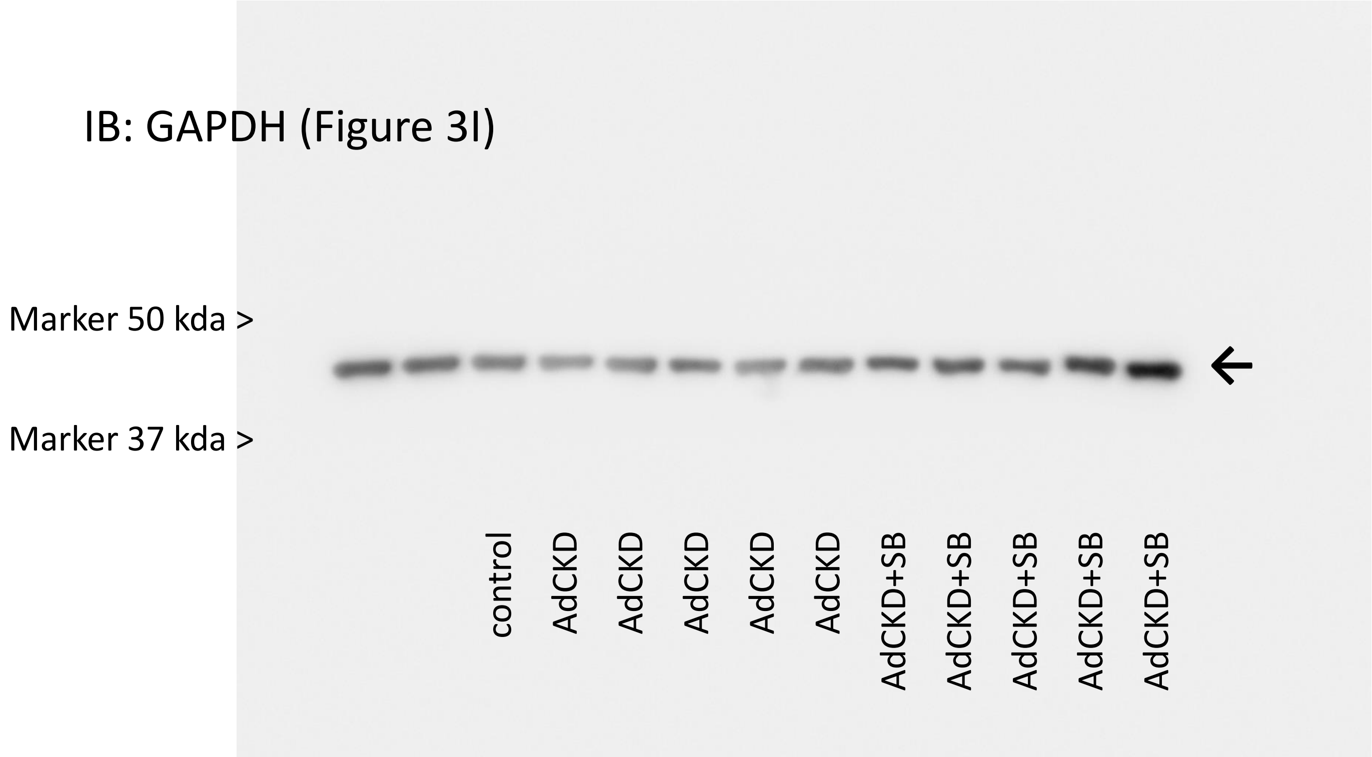

Supplementary Figure S4, Oshima, et al.

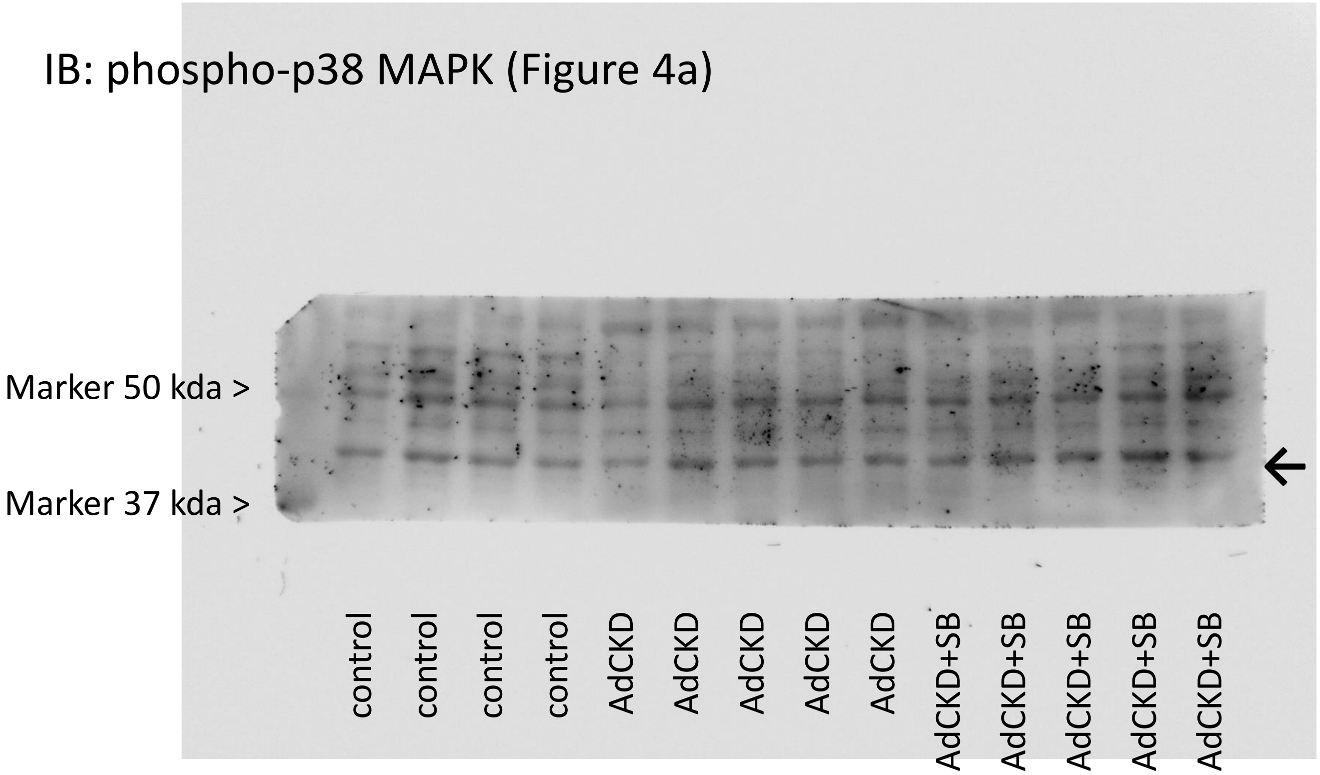

Supplementary Figure S5, Oshima, et al.

IB: phospho-JNK (Figure 4a)

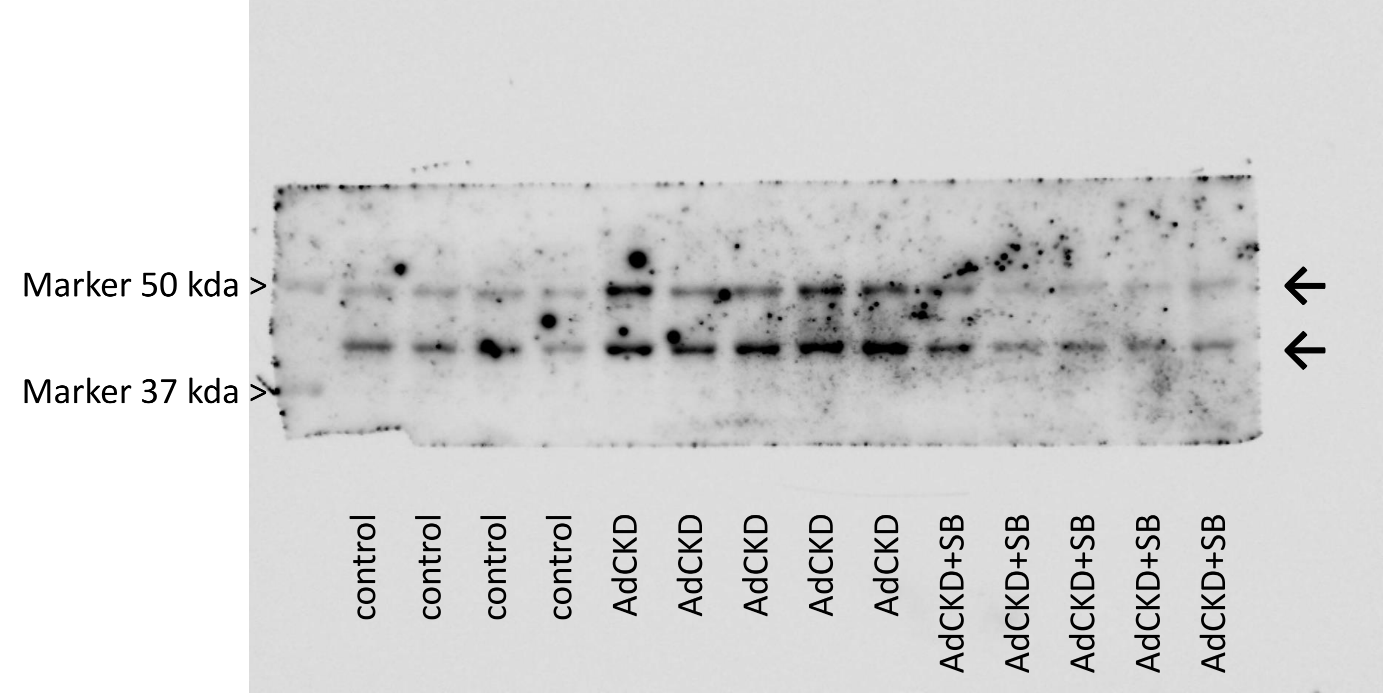

IB: phospho-JNK (Figure 4a)

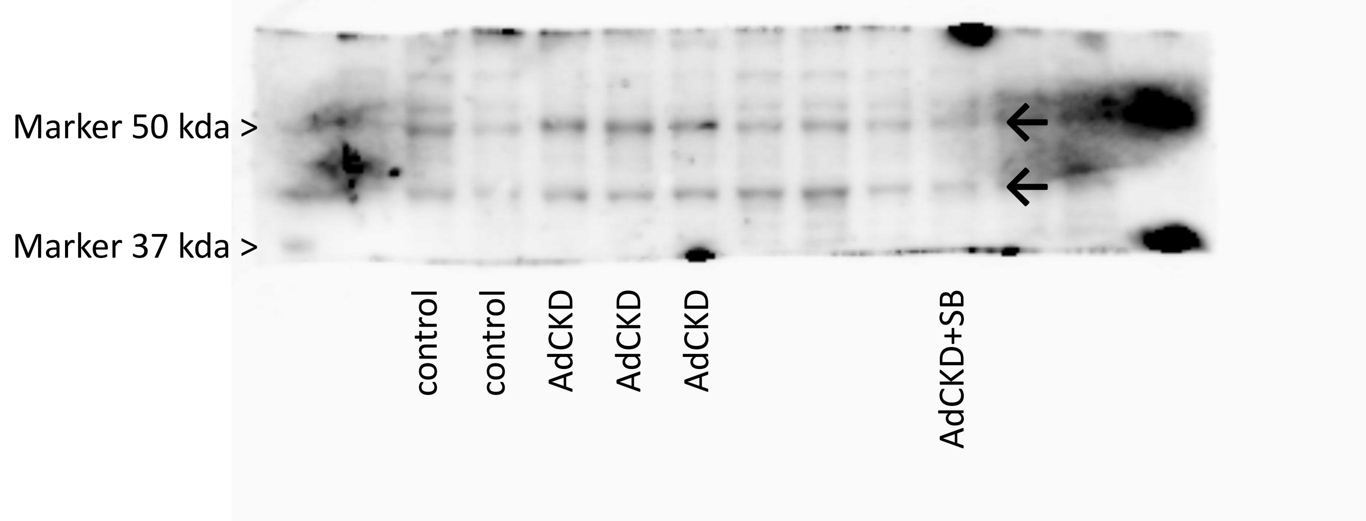

Supplementary Figure S6, Oshima, et al.

IB: phospho-ERK (Figure 4a)

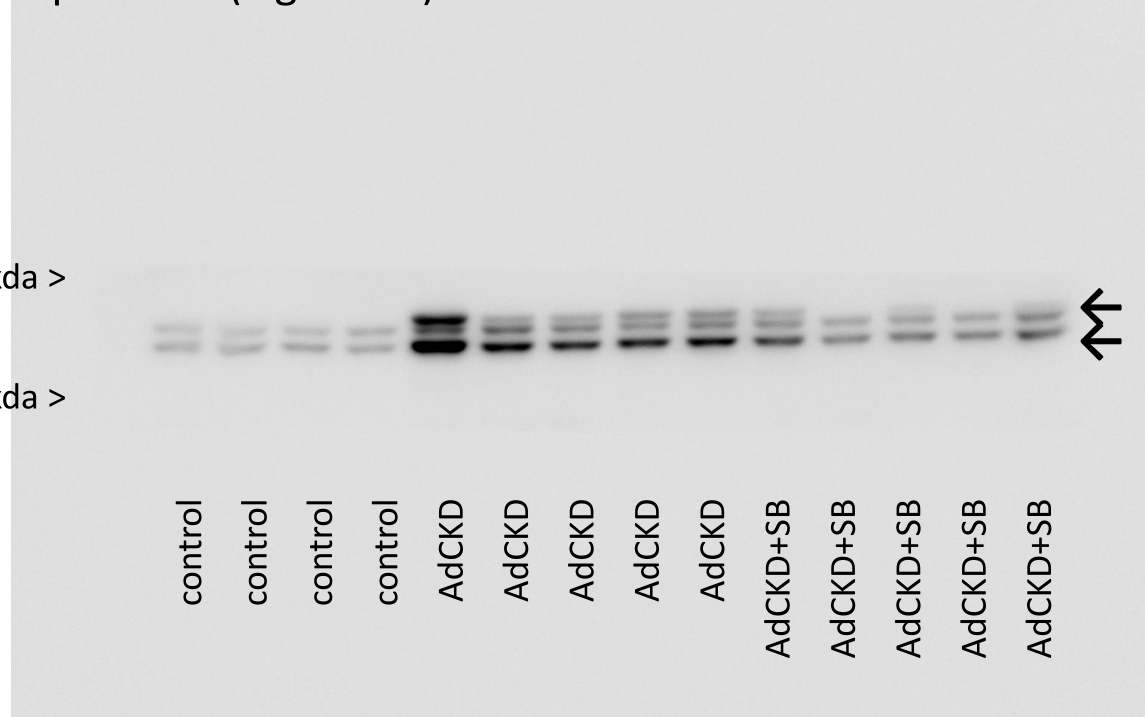

IB: phospho-ERK (Figure 4a)

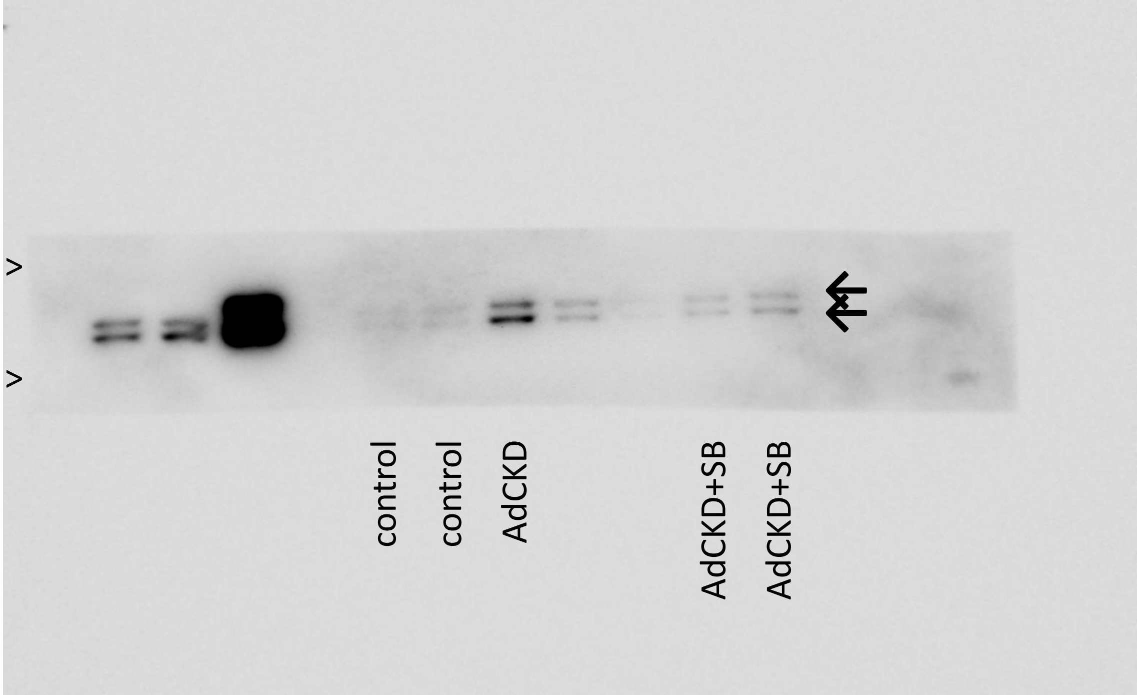

Supplementary Figure S7, Oshima, et al.

IB: phospho-ERK (Figure 4a)

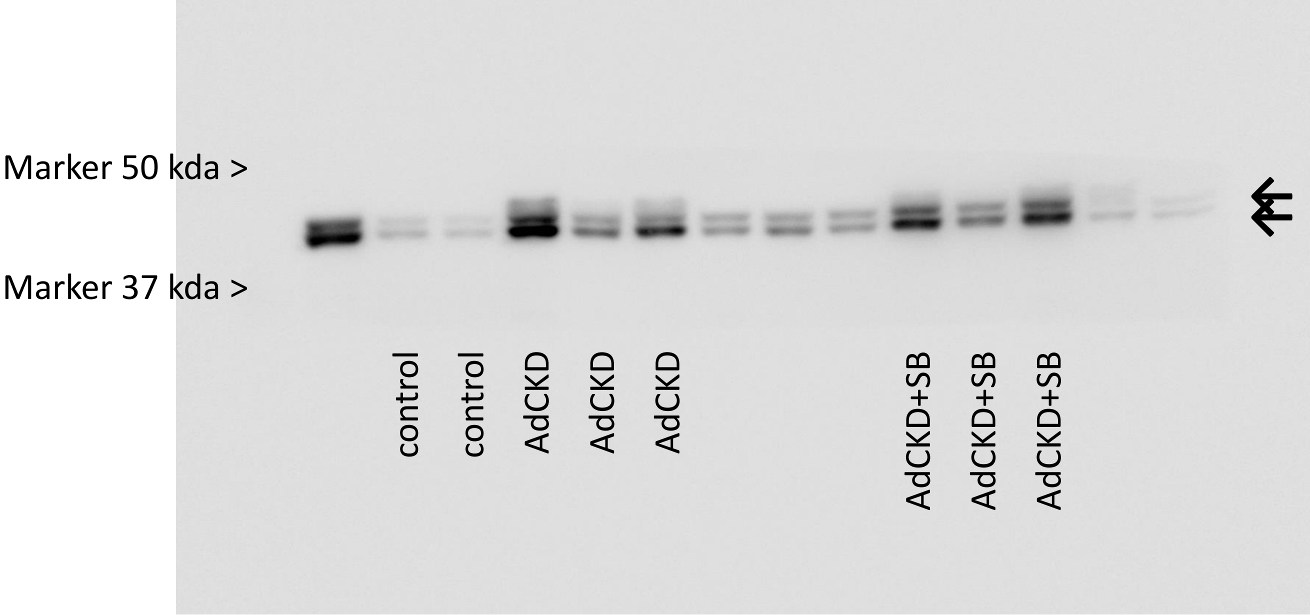

Supplementary Figure S8, Oshima, et al.

IB: phospho-Akt (Figure 4a)

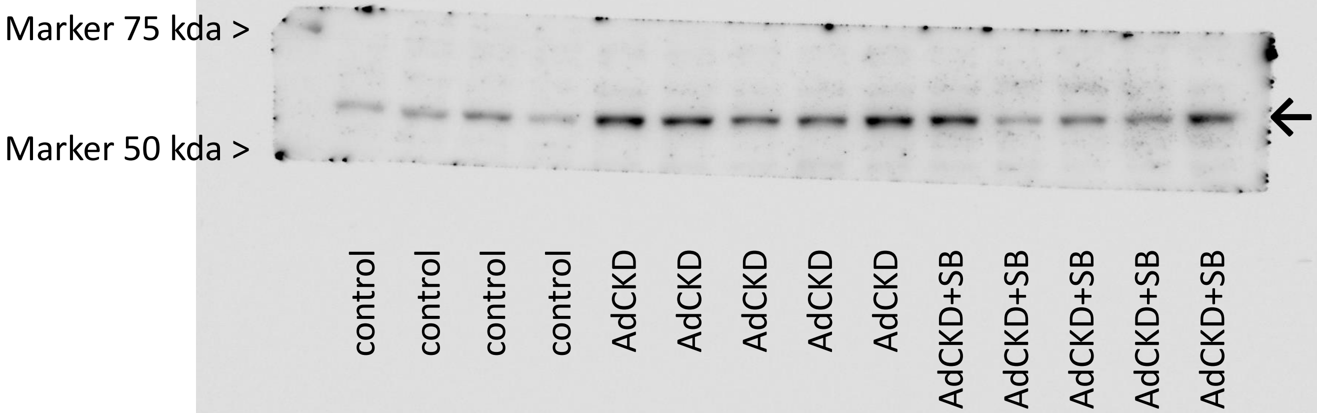

IB: phospho-Akt (Figure 4a)

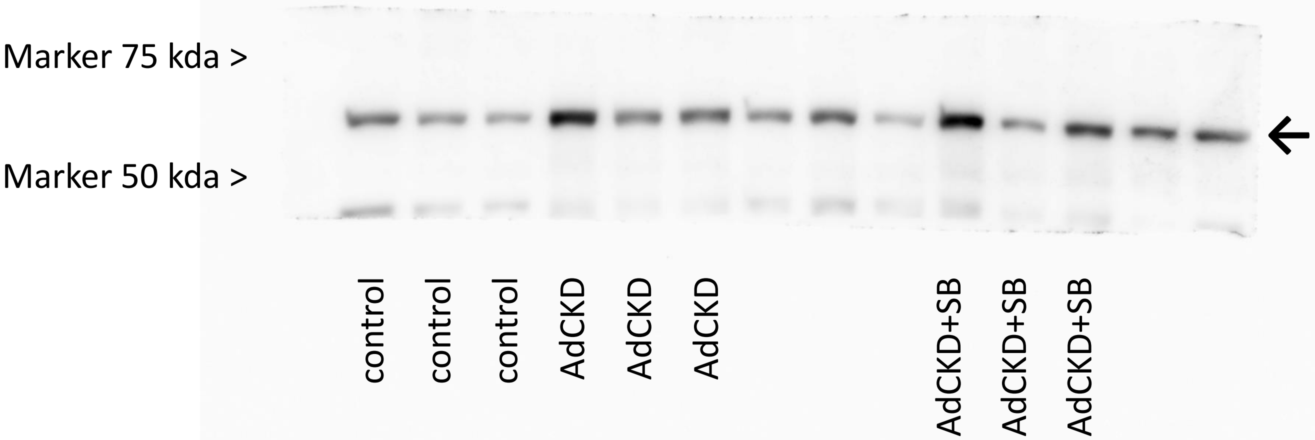

Supplementary Figure S9, Oshima, et al.

IB: phospho-Akt (Figure 4a)

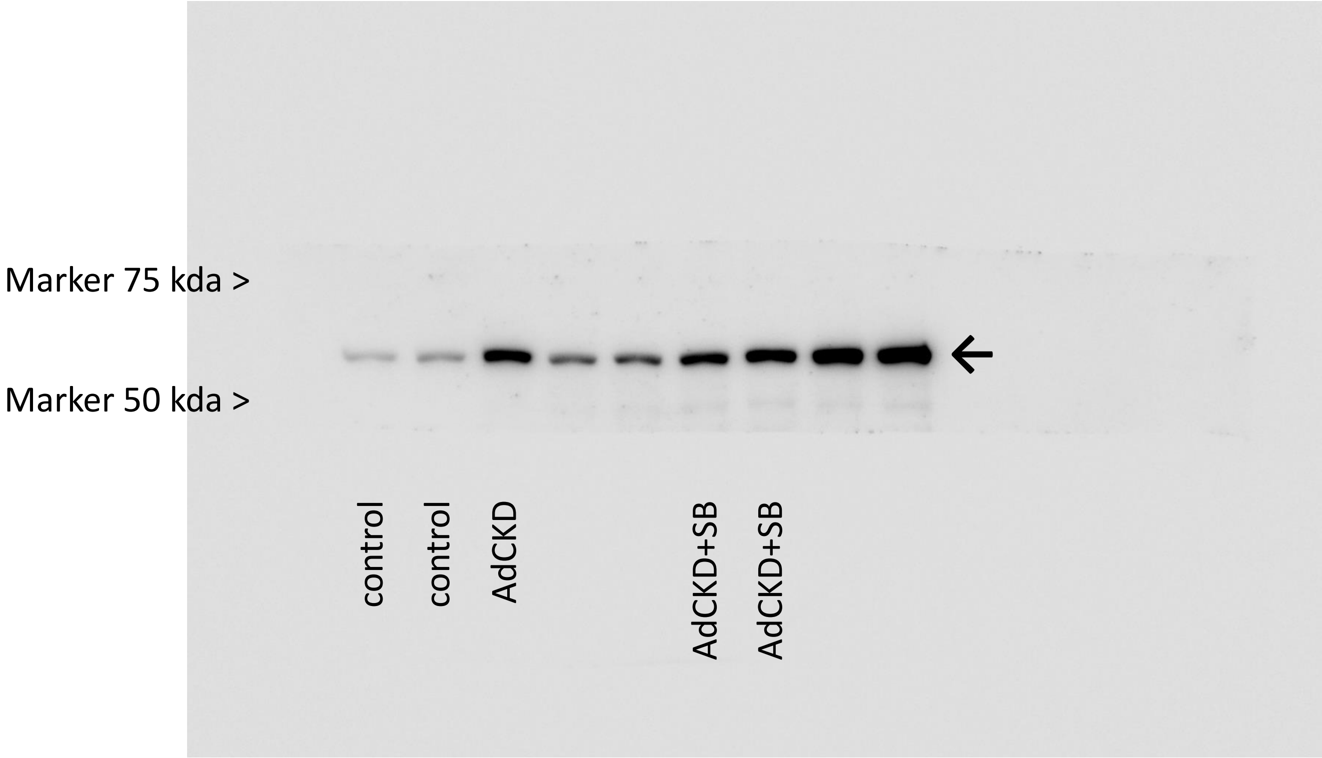

Supplementary Figure S10, Oshima, et al.

IB: phospho-NF-κB p65 (Figure 4a)

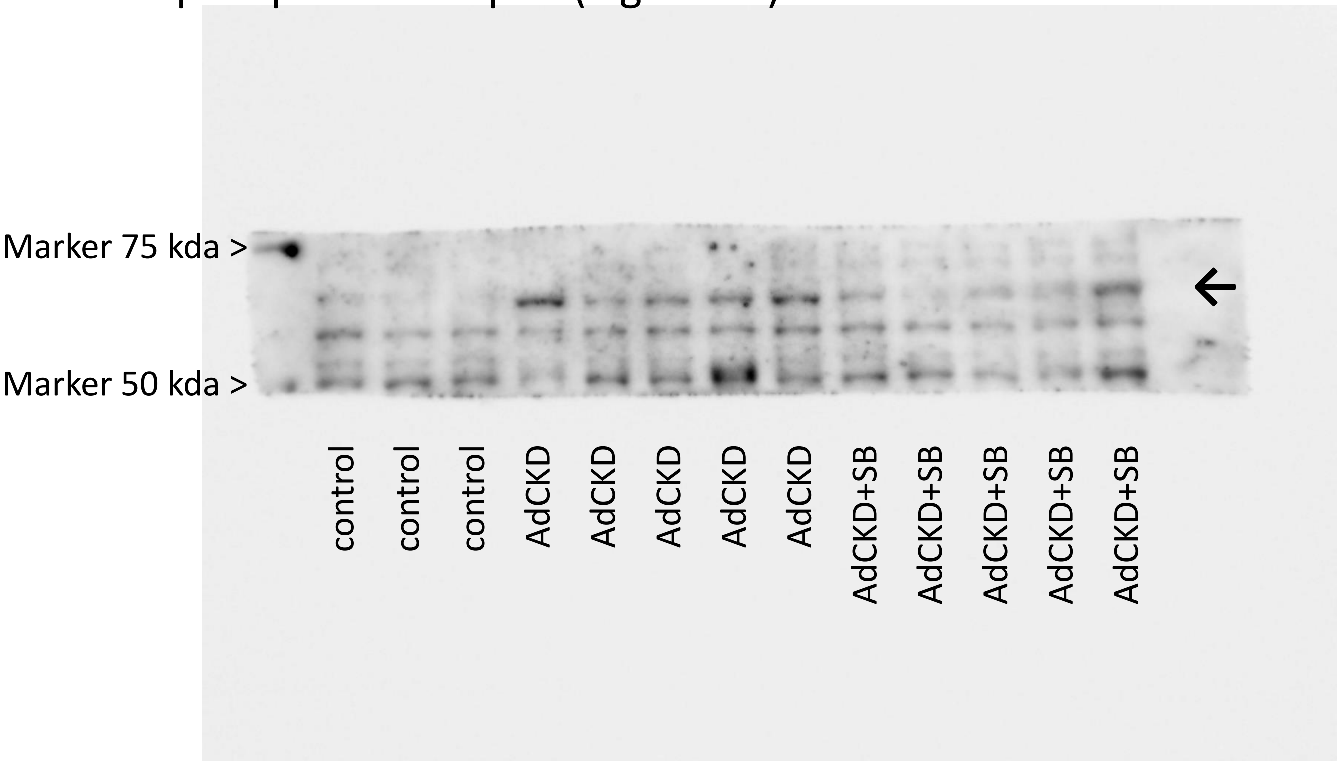

Supplementary Figure S11, Oshima, et al.

IB: phospho-NF-κB p65 (Figure 4a)

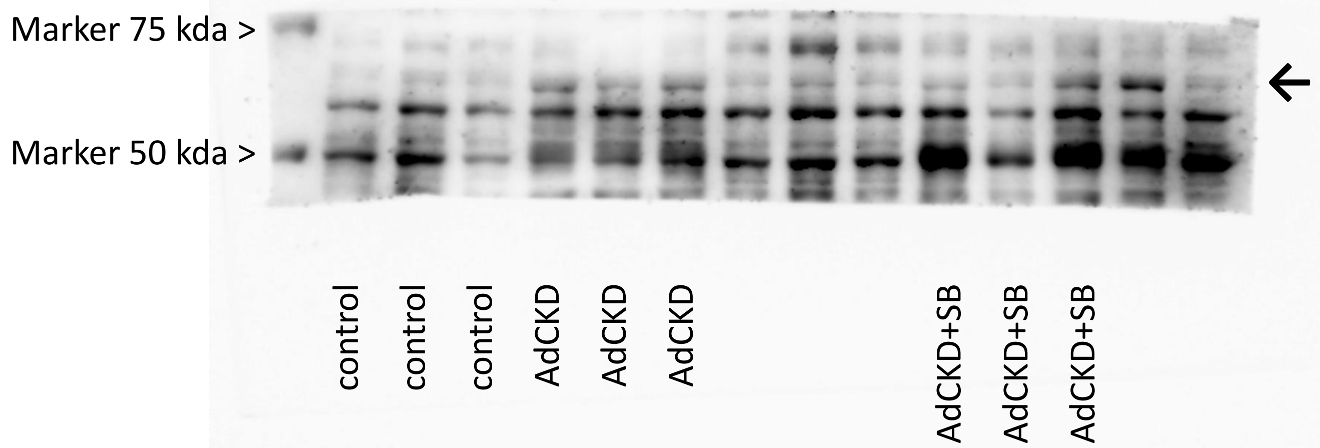

IB: phospho-NF-κB p65 (Figure 4a)

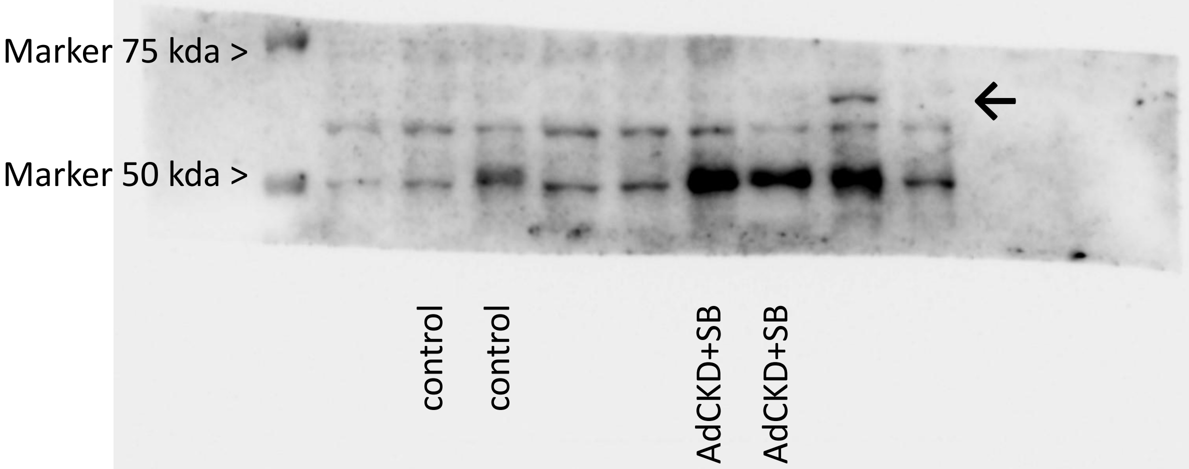

Supplementary Figure S12, Oshima, et al.

IB: βactin (Figure 4a)

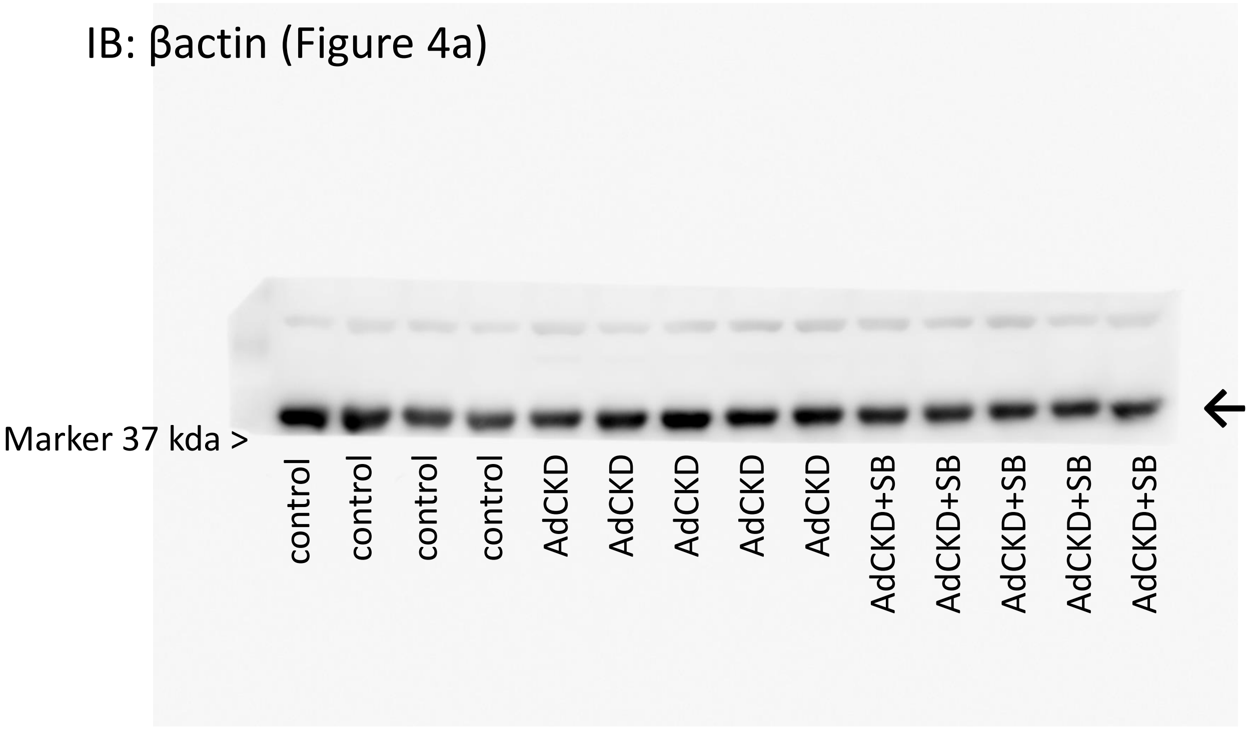

Supplementary Figure S13, Oshima, et al.

IB: phospho-p38MAPK (Figure 5i)

Marker 50 kda >

Marker 37 kda >

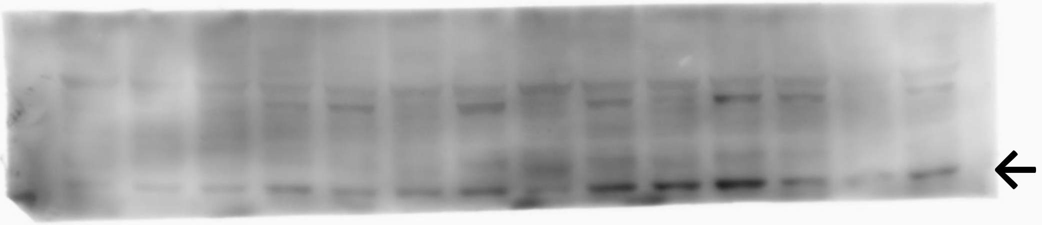

	1	2	3	4	5	6	1	2	3	4	5	6
1 µg/mL LPS	-	+	-	+	+	+	-	+	-	+	+	+
10 mM SB	-	-	+	+	-	-	-	-	+	+	-	-
10 µM U0126	-	-	-	-	+	-	-	-	-	-	+	-
50 µM SP600125	-	-	-	-	-	+	-	-	-	-	-	+

IB: phospho-p38MAPK (Figure 5i)

Marker 50 kda >

Marker 37 kda >

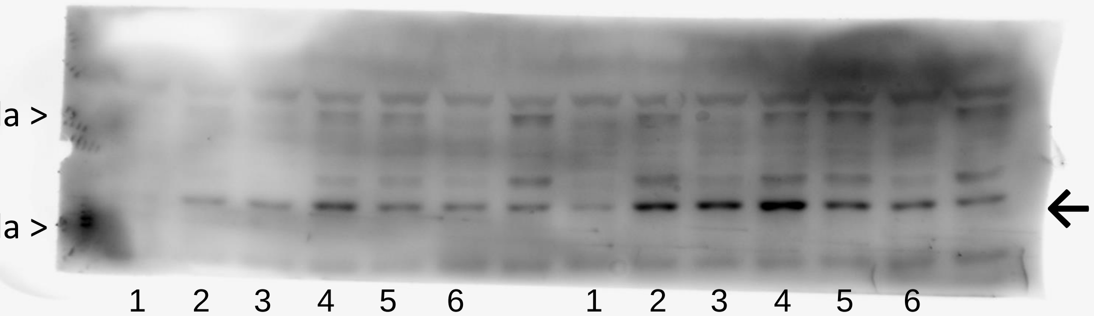

	1	2	3	4	5	6	1	2	3	4	5	6
1 µg/mL LPS	-	+	-	+	+	+	-	+	-	+	+	+
10 mM SB	-	-	+	+	-	-	-	-	+	+	-	-
10 µM U0126	-	-	-	-	+	-	-	-	-	-	+	-
50 µM SP600125	-	-	-	-	-	+	-	-	-	-	-	+

Supplementary Figure S14, Oshima, et al.

IB: phospho-p38MAPK (Figure 5i)

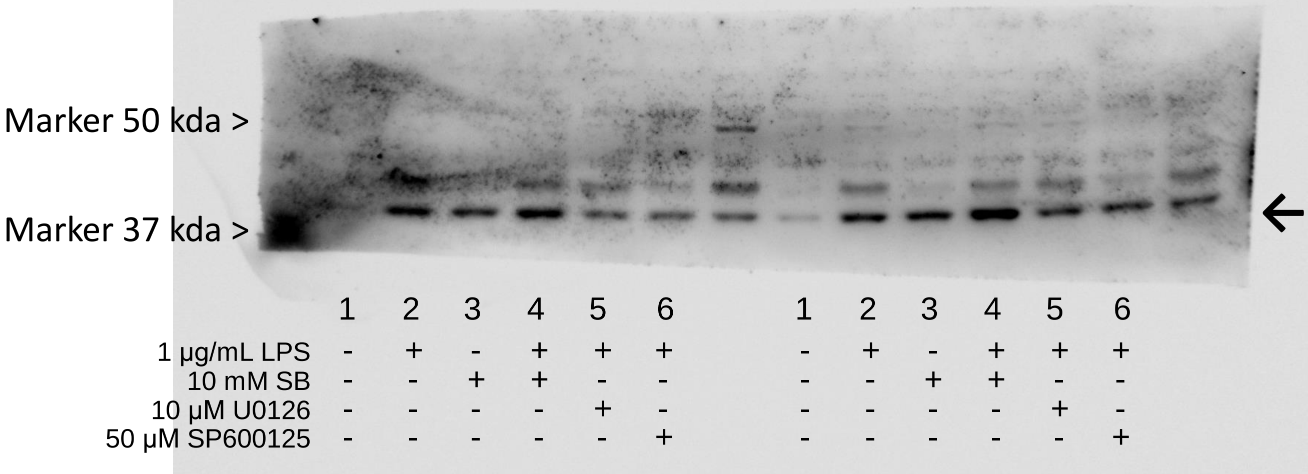

IB: phospho-JNK (Figure 5i)

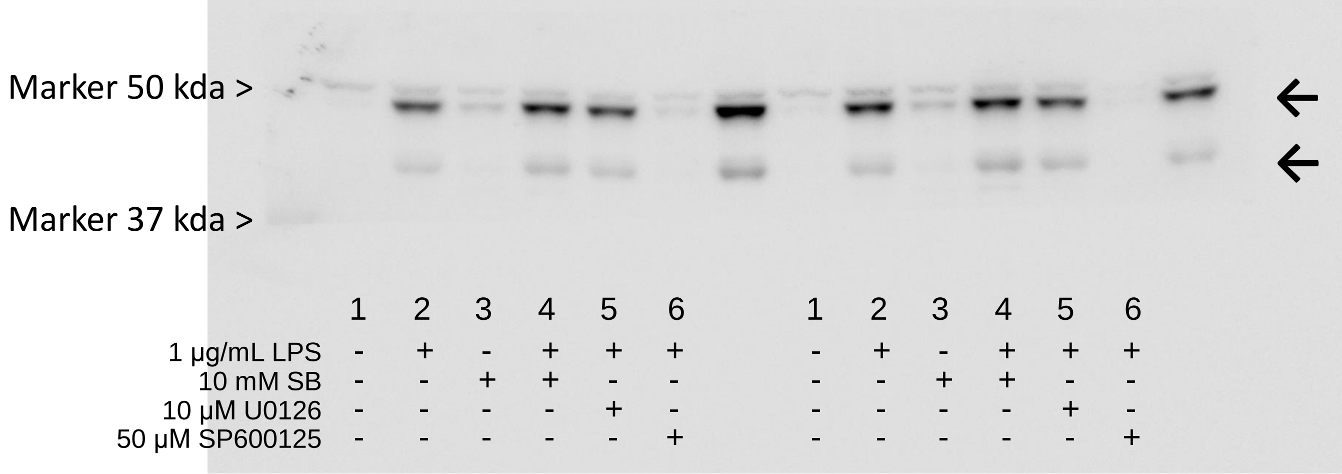

Supplementary Figure S15, Oshima, et al.

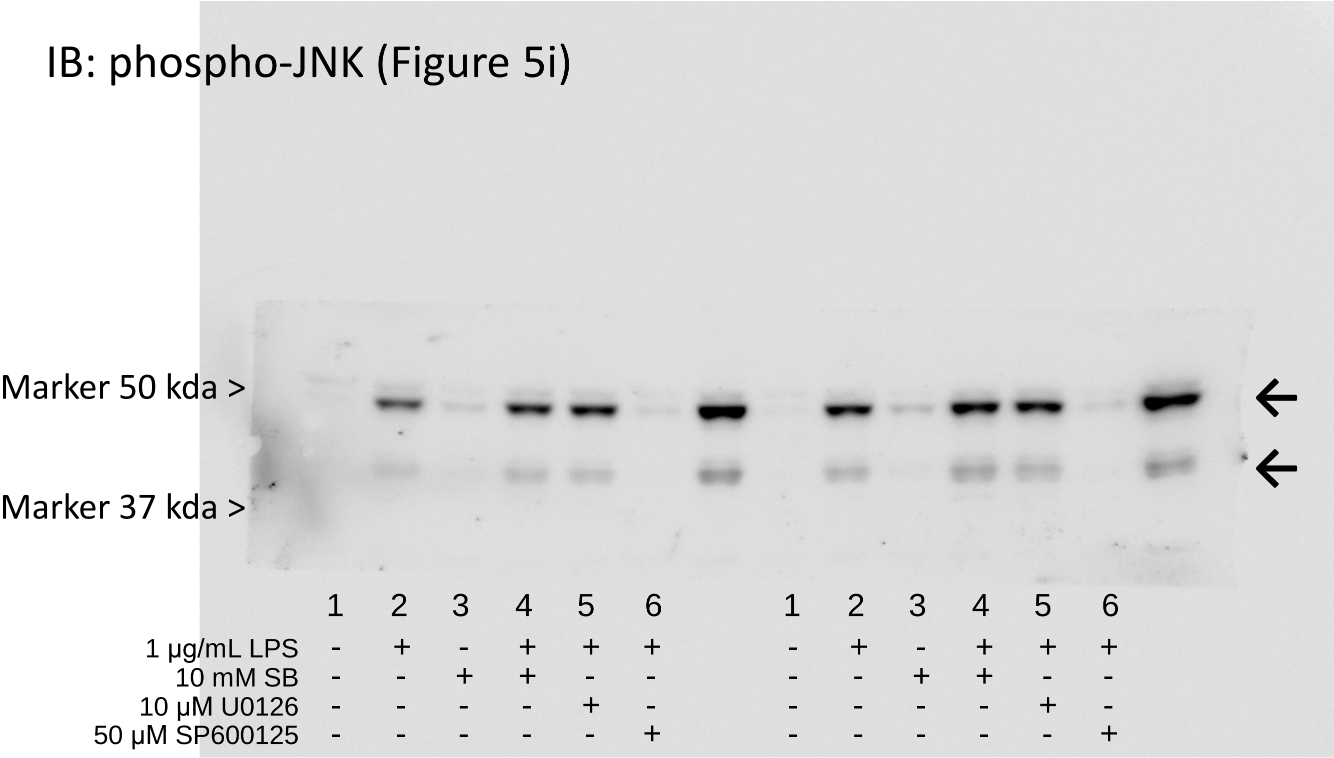

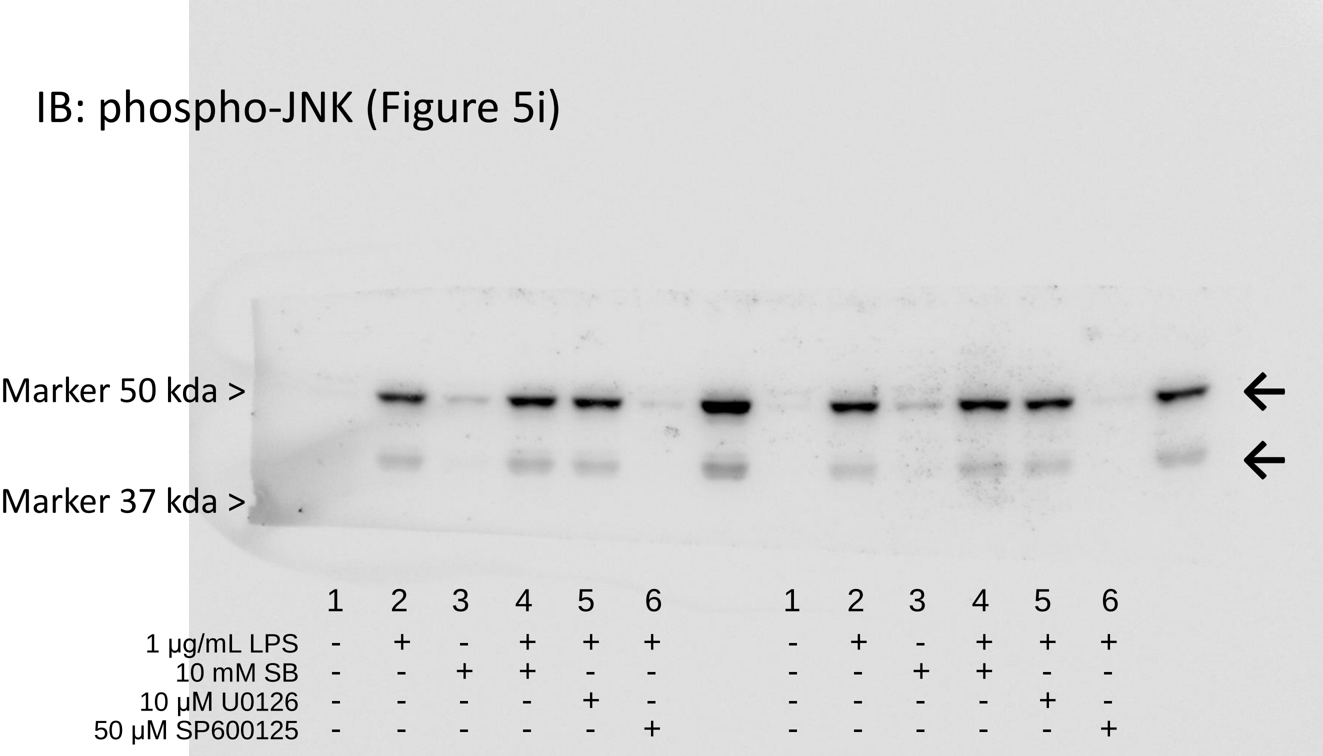

Supplementary Figure S16, Oshima, et al.

IB: phospho-ERK (Figure 5i)

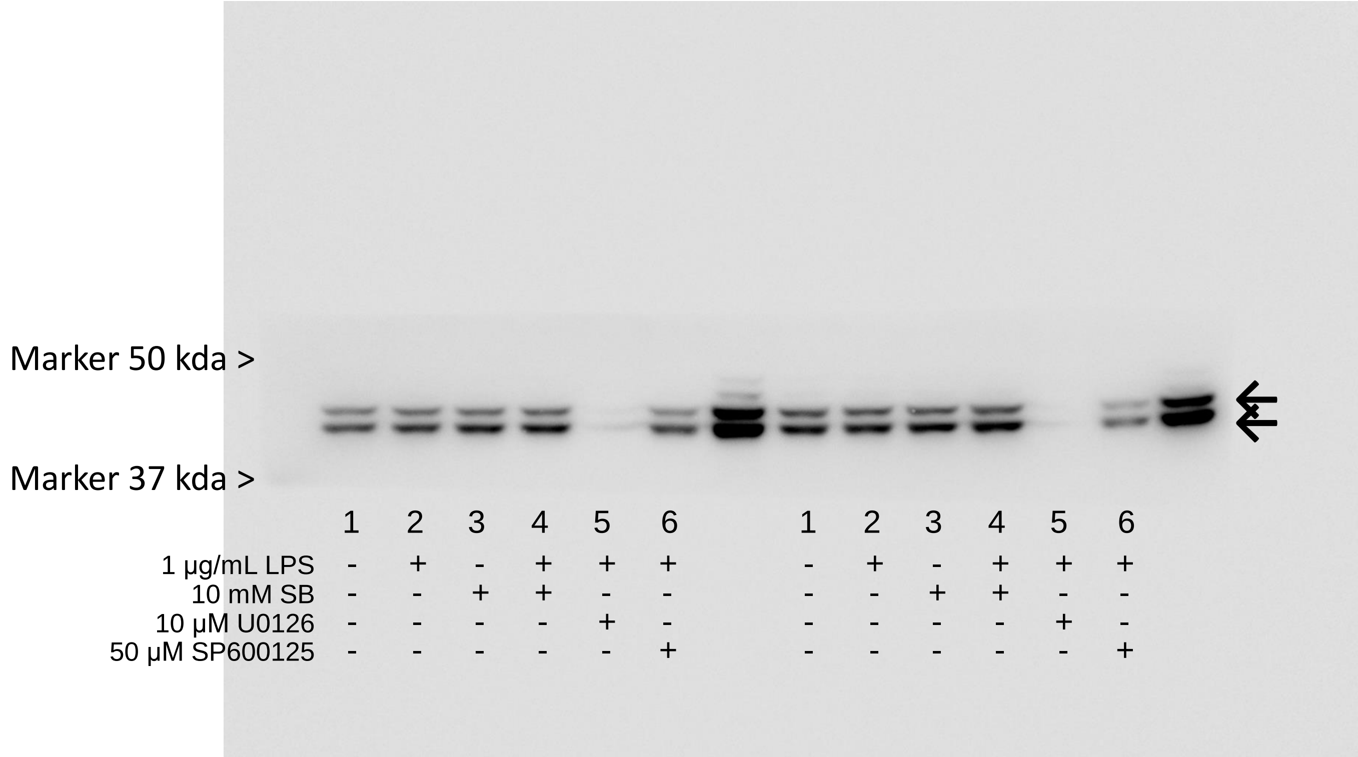

IB: phospho-ERK (Figure 5i)

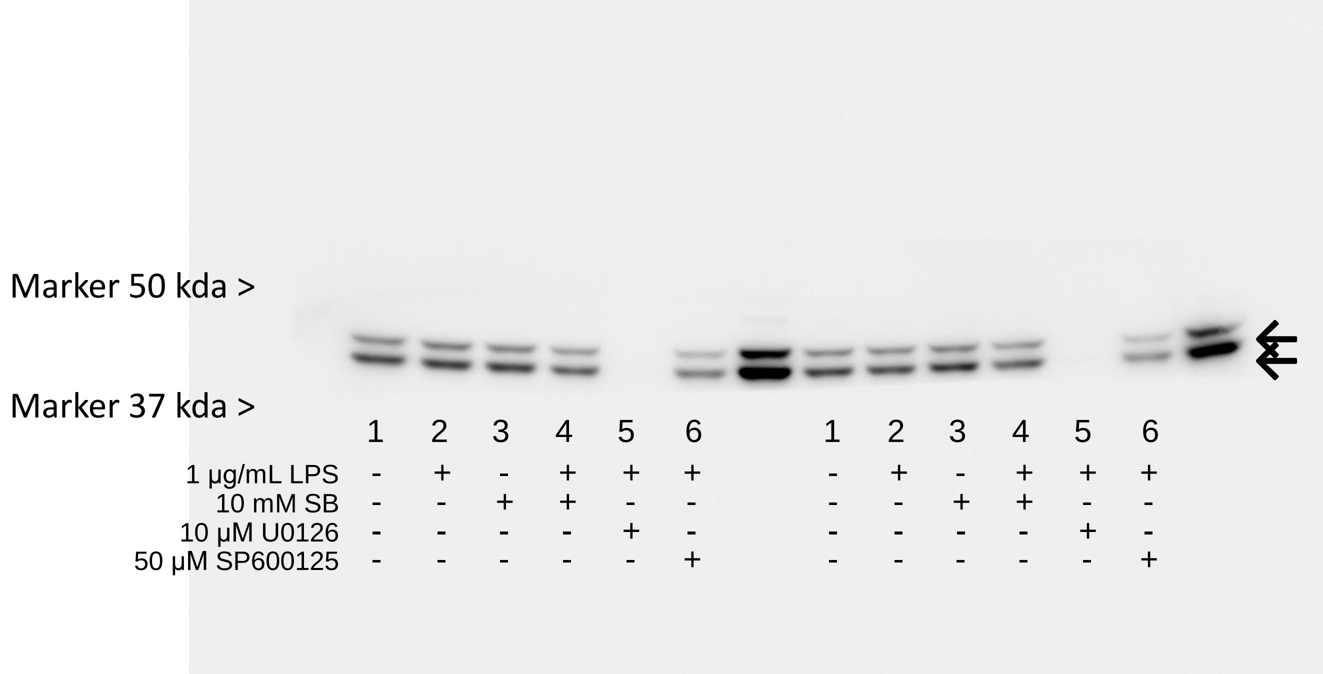

Supplementary Figure S17, Oshima, et al.

IB: phospho-ERK (Figure 5i)

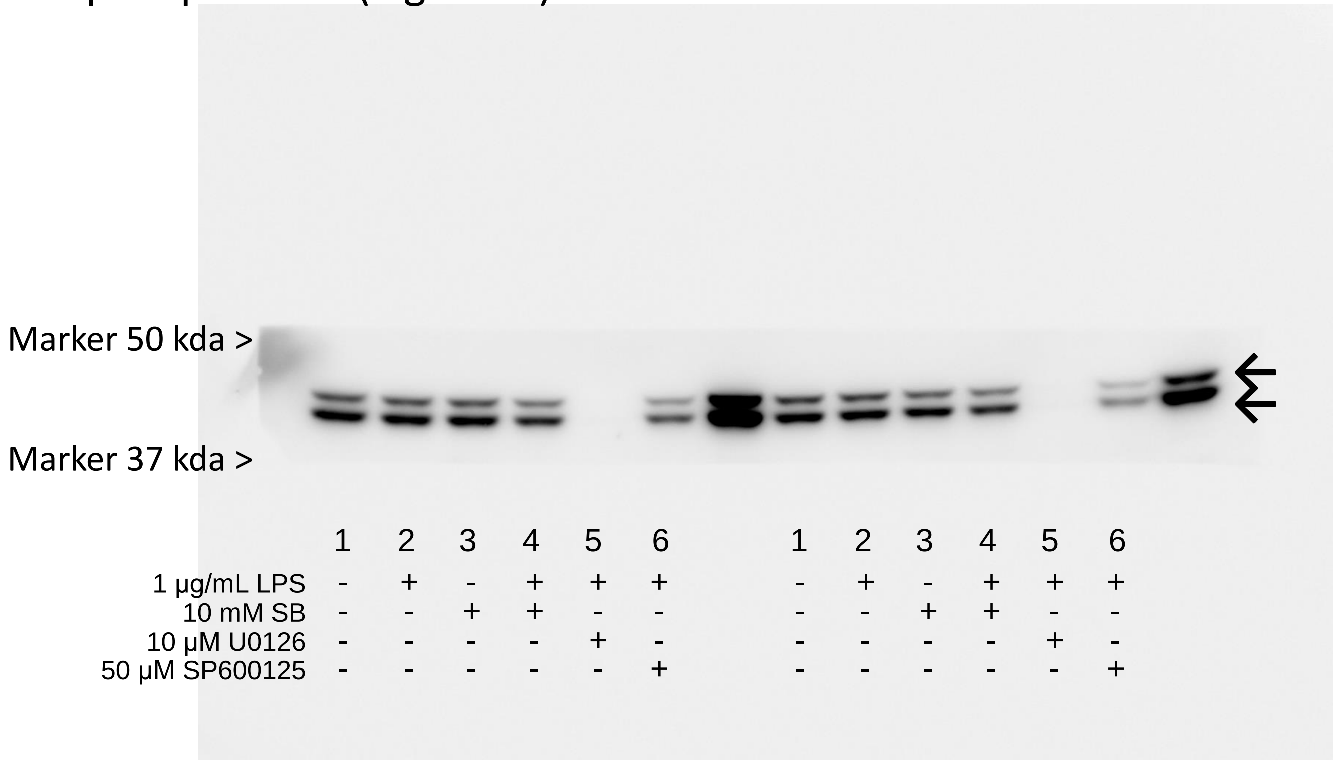

IB: phospho-Akt (Figure 5i)

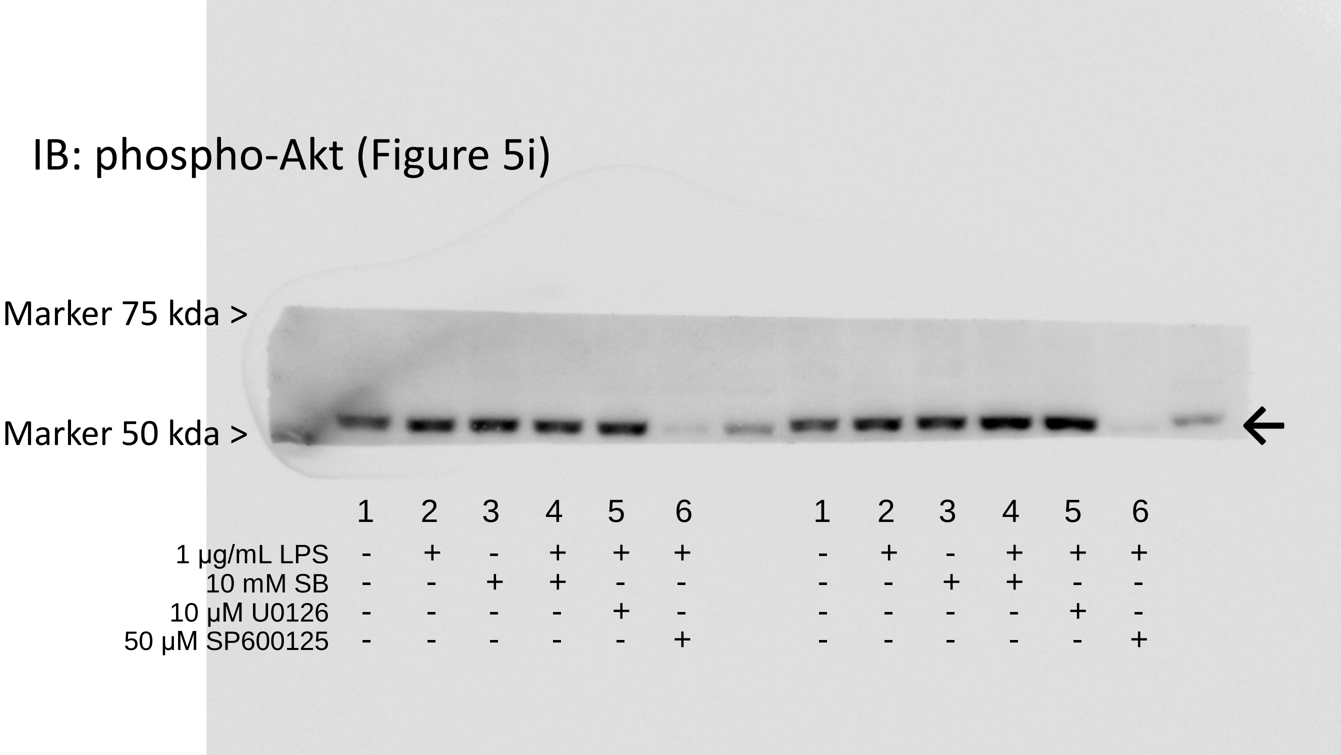

Supplementary Figure S18, Oshima, et al.

IB: phospho-Akt (Figure 5i)

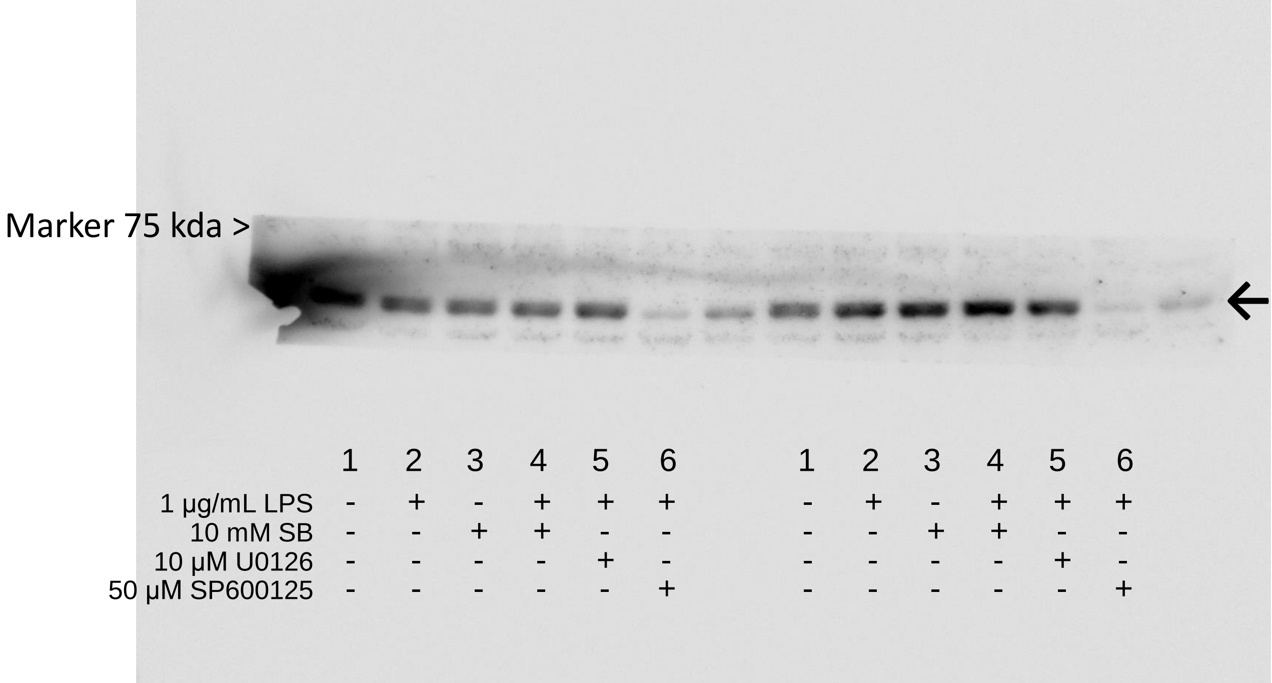

IB: phospho-Akt (Figure 5i)

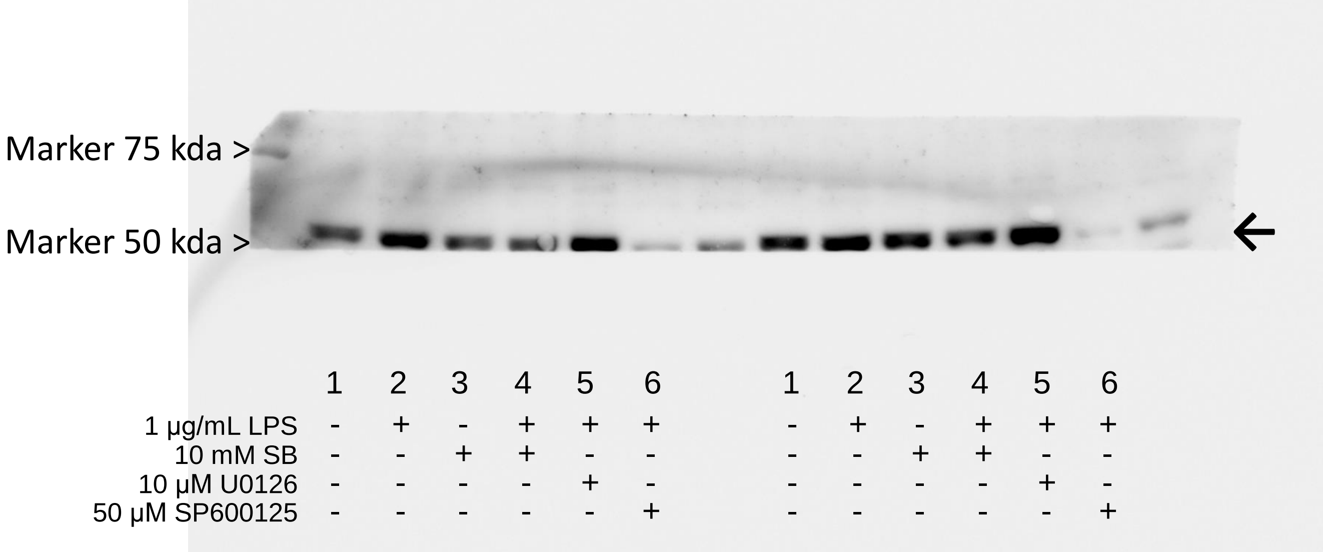

Supplementary Figure S19, Oshima, et al.

IB: phospho-NF-κB p65 (Figure 5i)

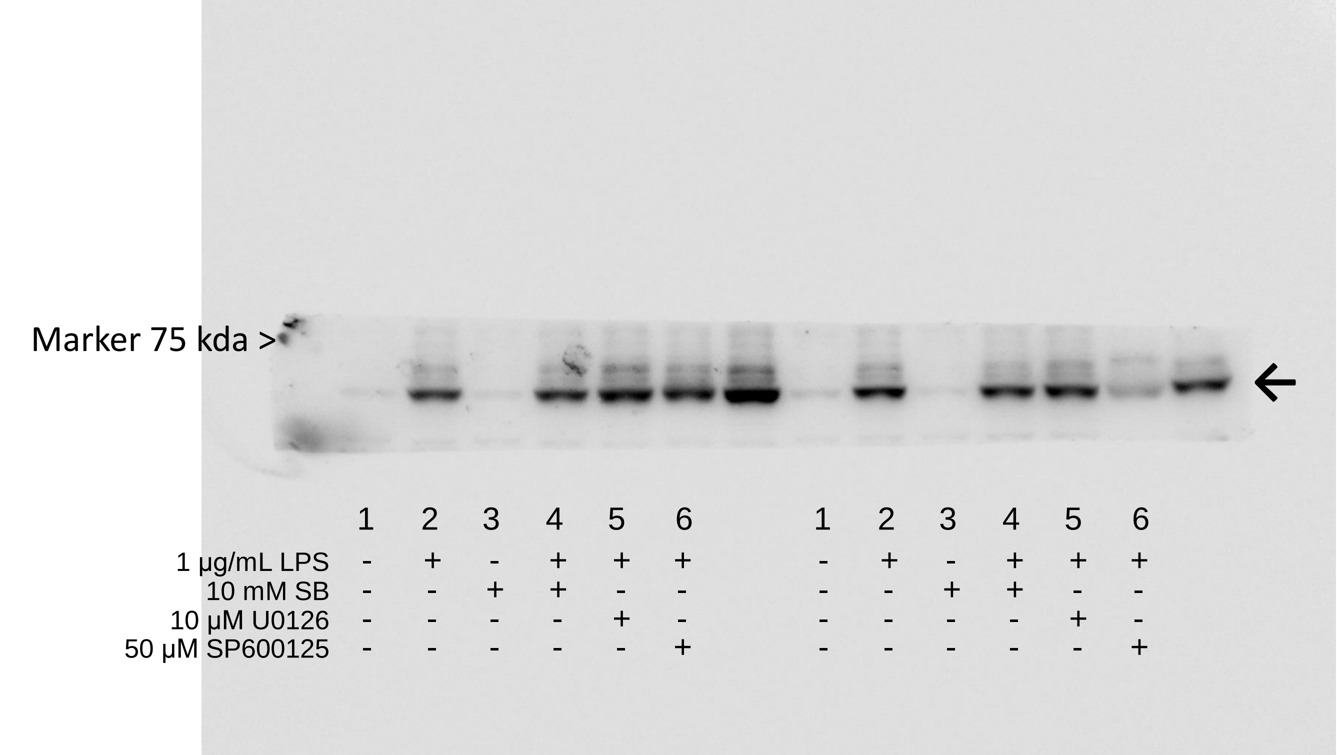

IB: phospho-NF-κB p65 (Figure 5i)

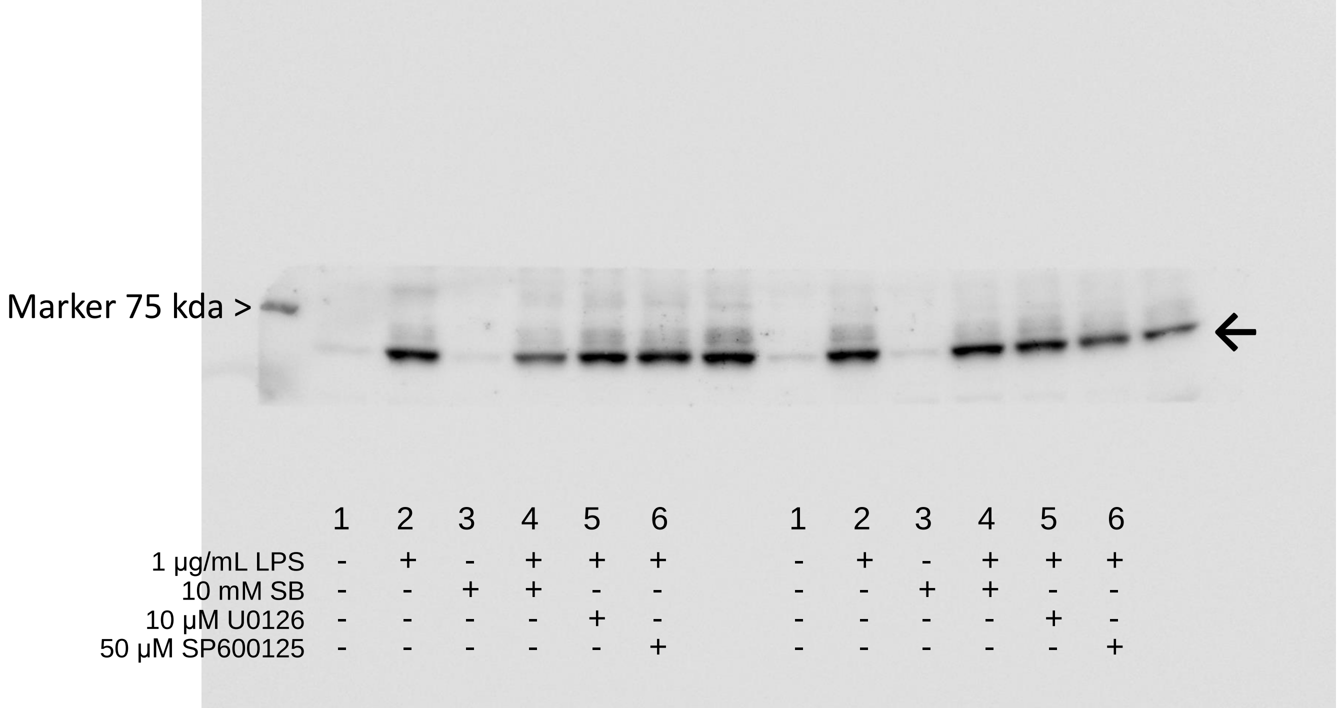

Supplementary Figure S20, Oshima, et al.

IB: phospho-NF-κB p65 (Figure 5i)

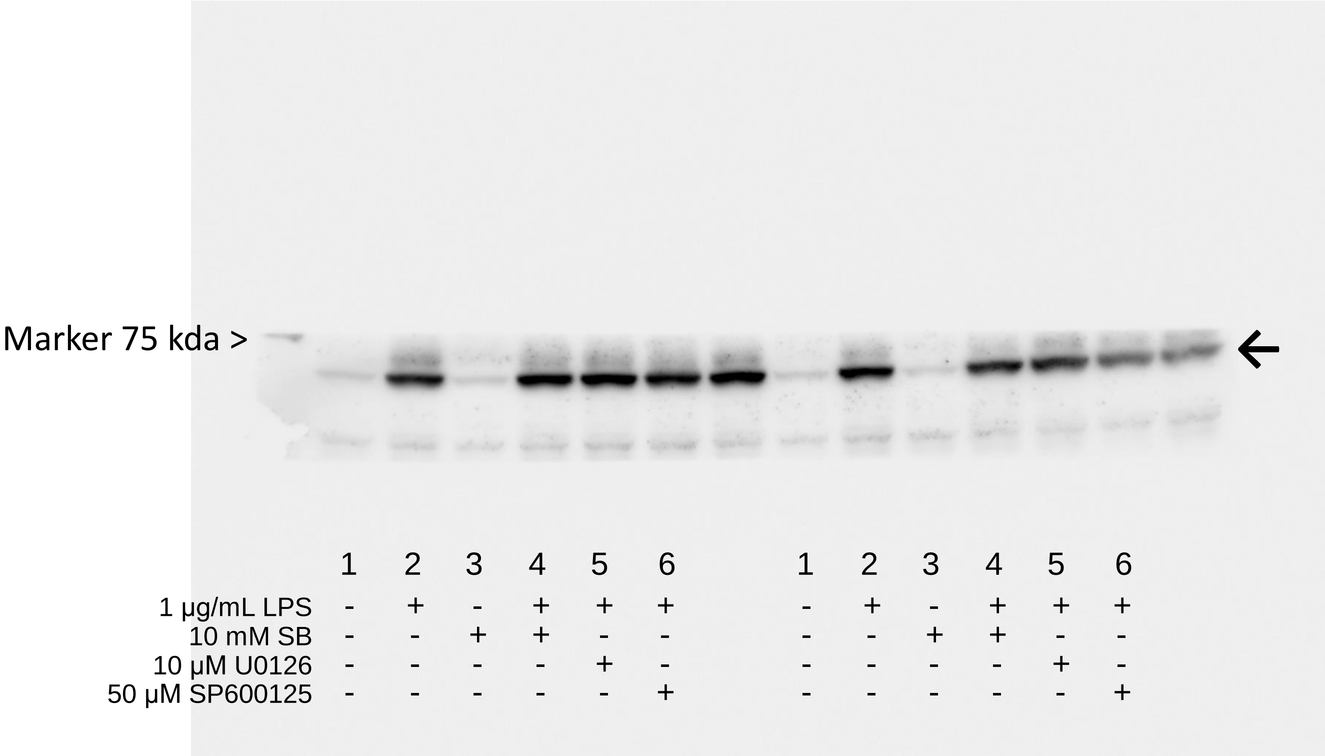

IB: βactin (Figure 5i)

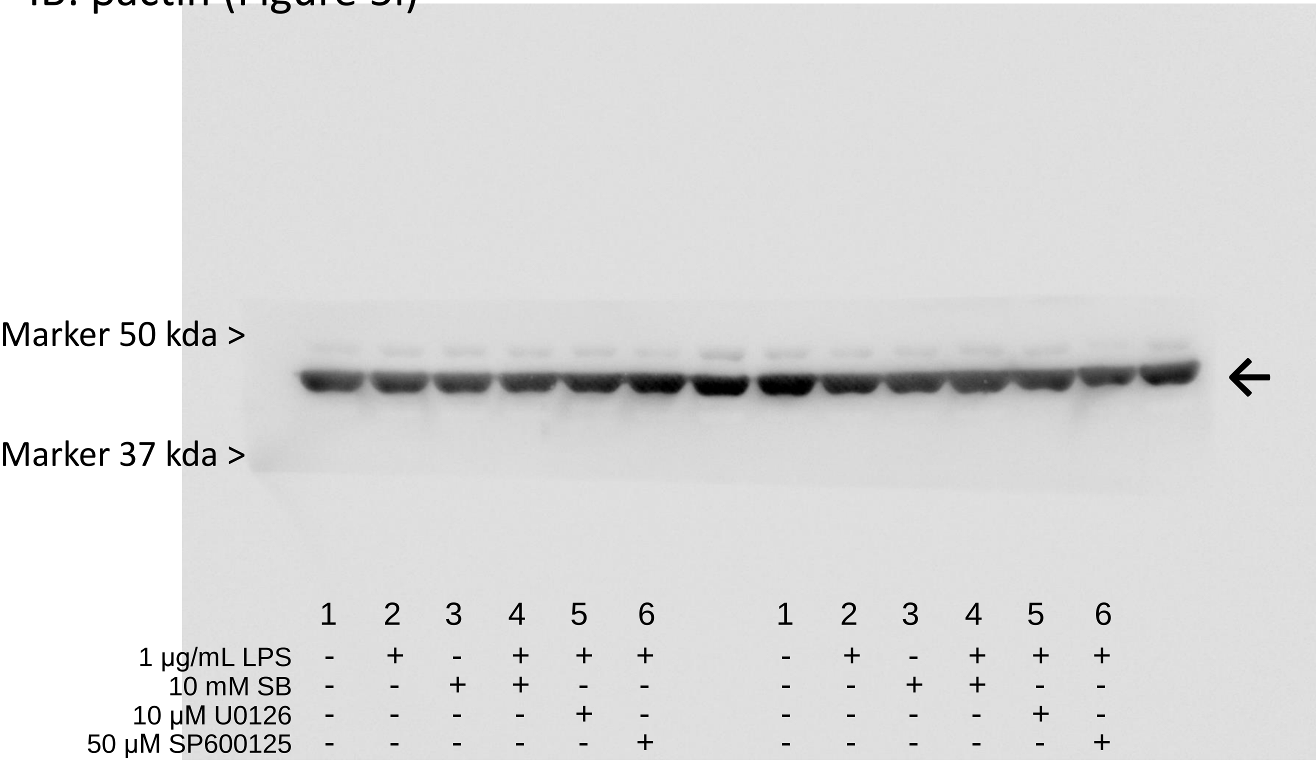

Supplementary Figure S21, Oshima, et al.

IB: βactin (Figure 5i)

Marker 50 kda >

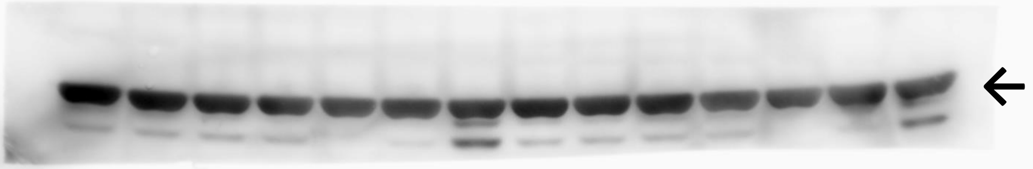

Marker 37 kda >

	1	2	3	4	5	6	1	2	3	4	5	6
1 µg/mL LPS	-	+	-	+	+	+	-	+	-	+	+	+
10 mM SB	-	-	+	+	-	-	-	-	+	+	-	-
10 µM U0126	-	-	-	-	+	-	-	-	-	-	+	-
50 µM SP600125	-	-	-	-	-	+	-	-	-	-	-	+

IB: βactin (Figure 5i)

Marker 50 kda >

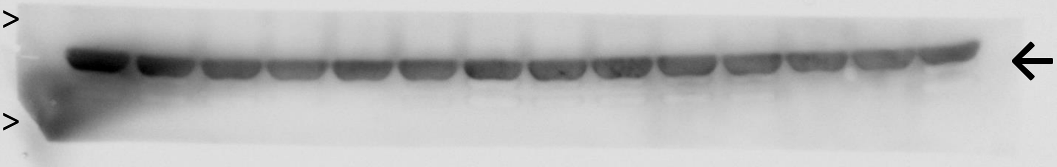

Marker 37 kda >

	1	2	3	4	5	6	1	2	3	4	5	6
1 µg/mL LPS	-	+	-	+	+	+	-	+	-	+	+	+
10 mM SB	-	-	+	+	-	-	-	-	+	+	-	-
10 µM U0126	-	-	-	-	+	-	-	-	-	-	+	-
50 µM SP600125	-	-	-	-	-	+	-	-	-	-	-	+

Supplementary Figure S22, Oshima, et al.

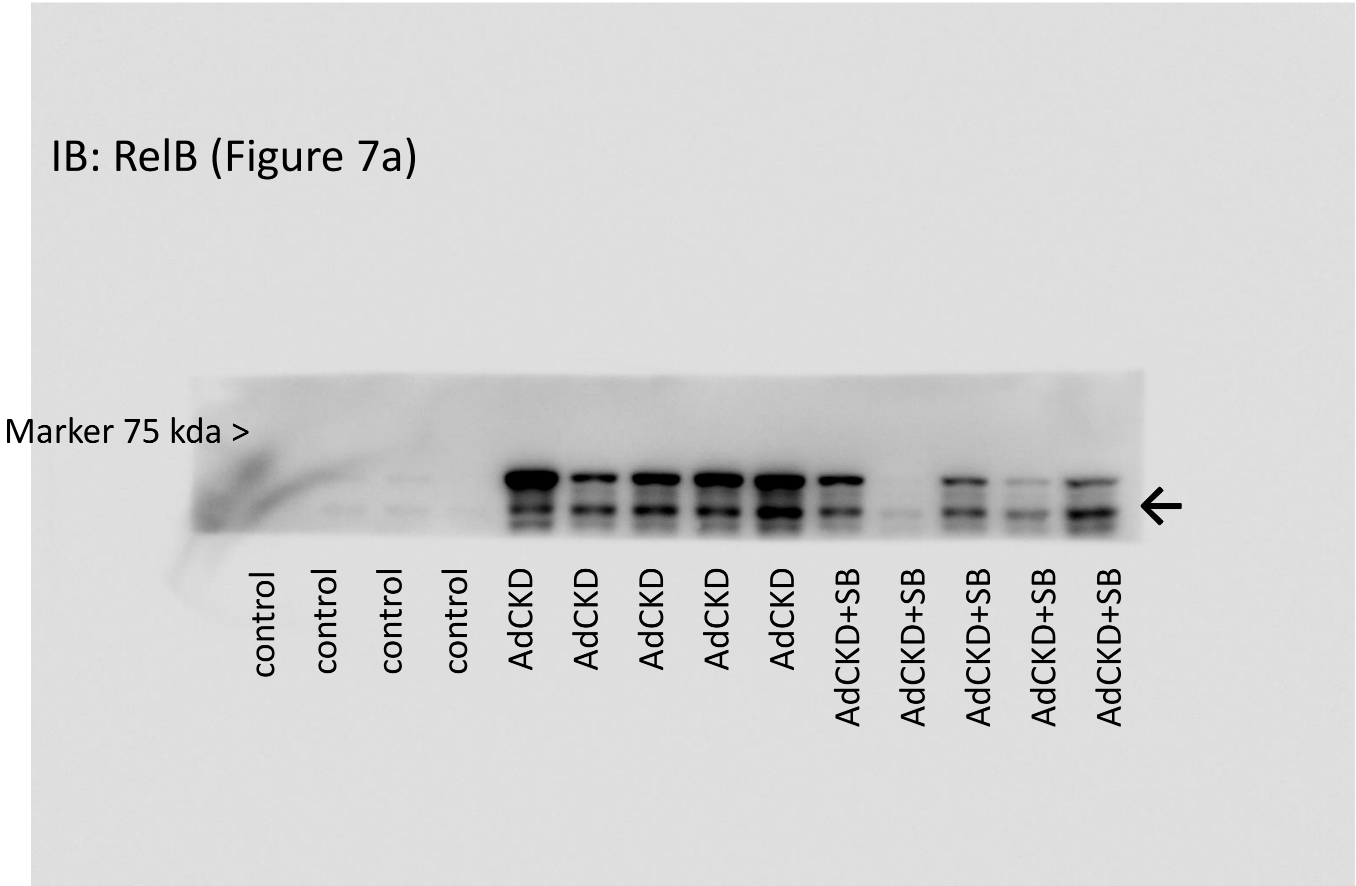

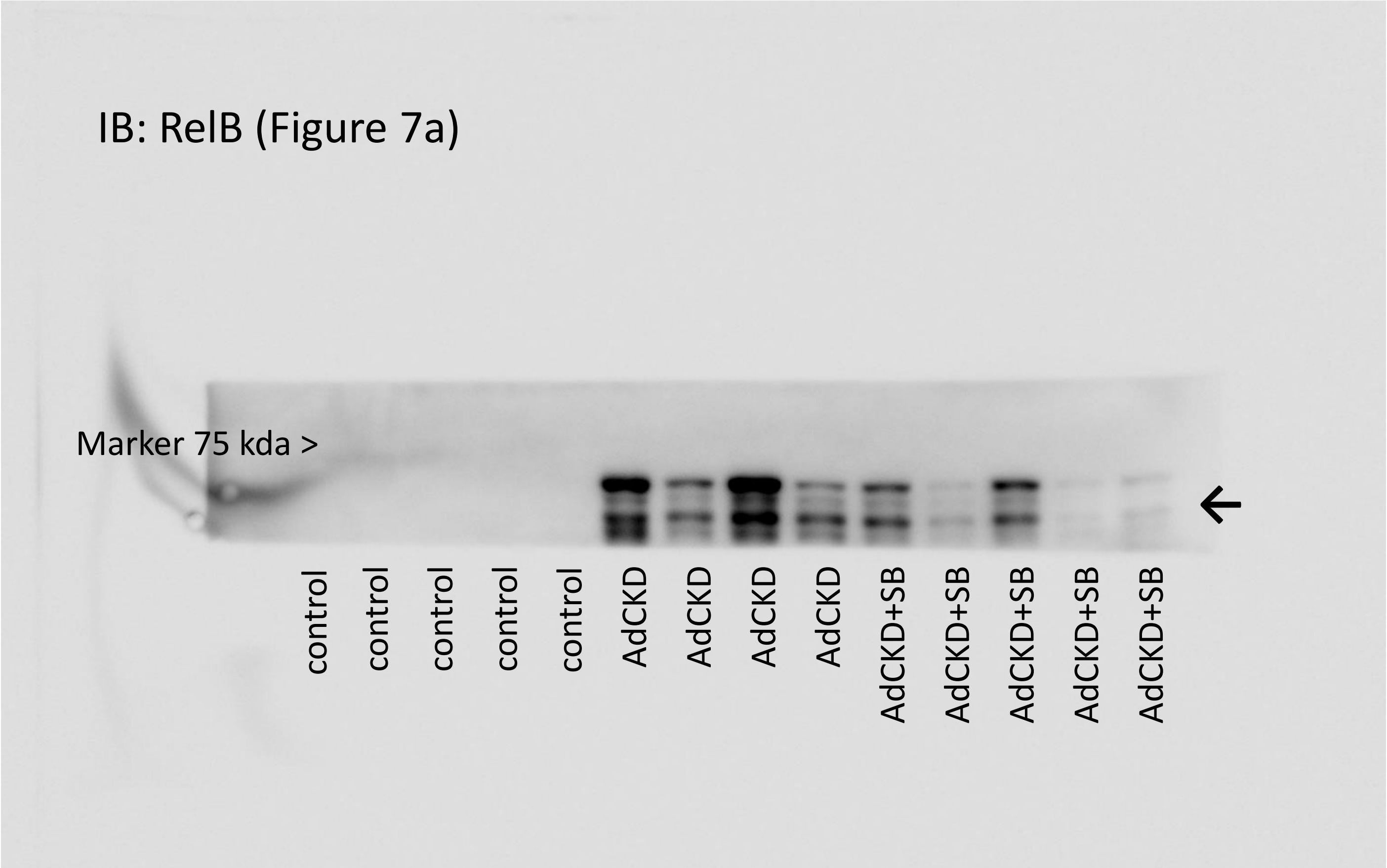

Supplementary Figure S23, Oshima, et al.

IB: βactin (Figure 4a)

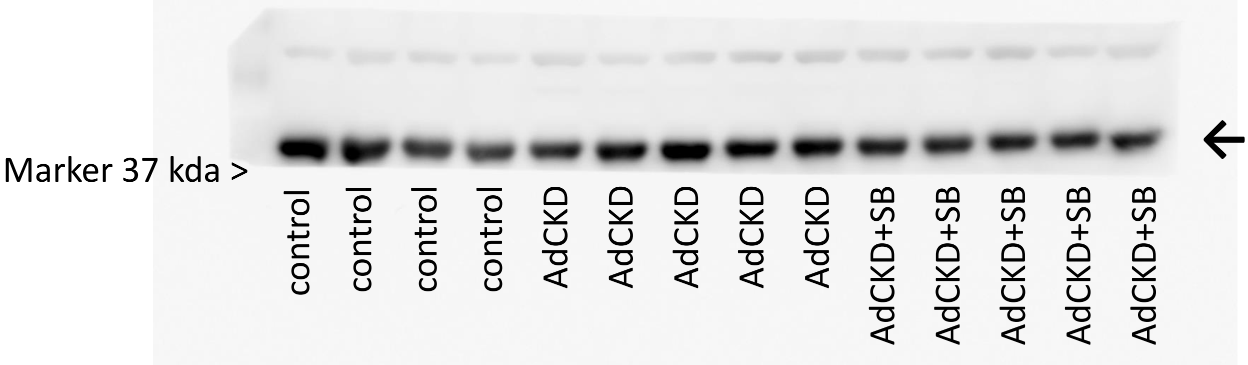

IB : βactin (Figure 7a)

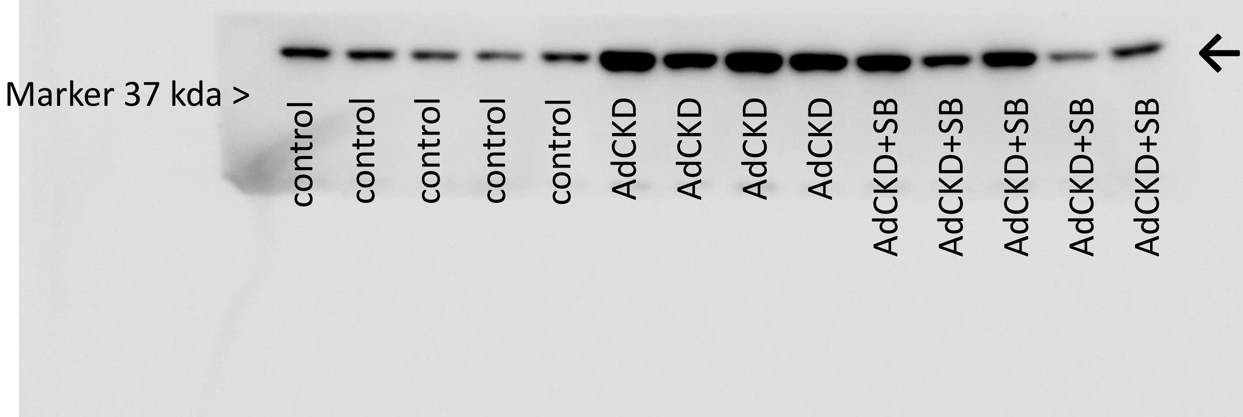

Supplementary Figure S24, Oshima, et al.

IB: RelB (Figure 7b)

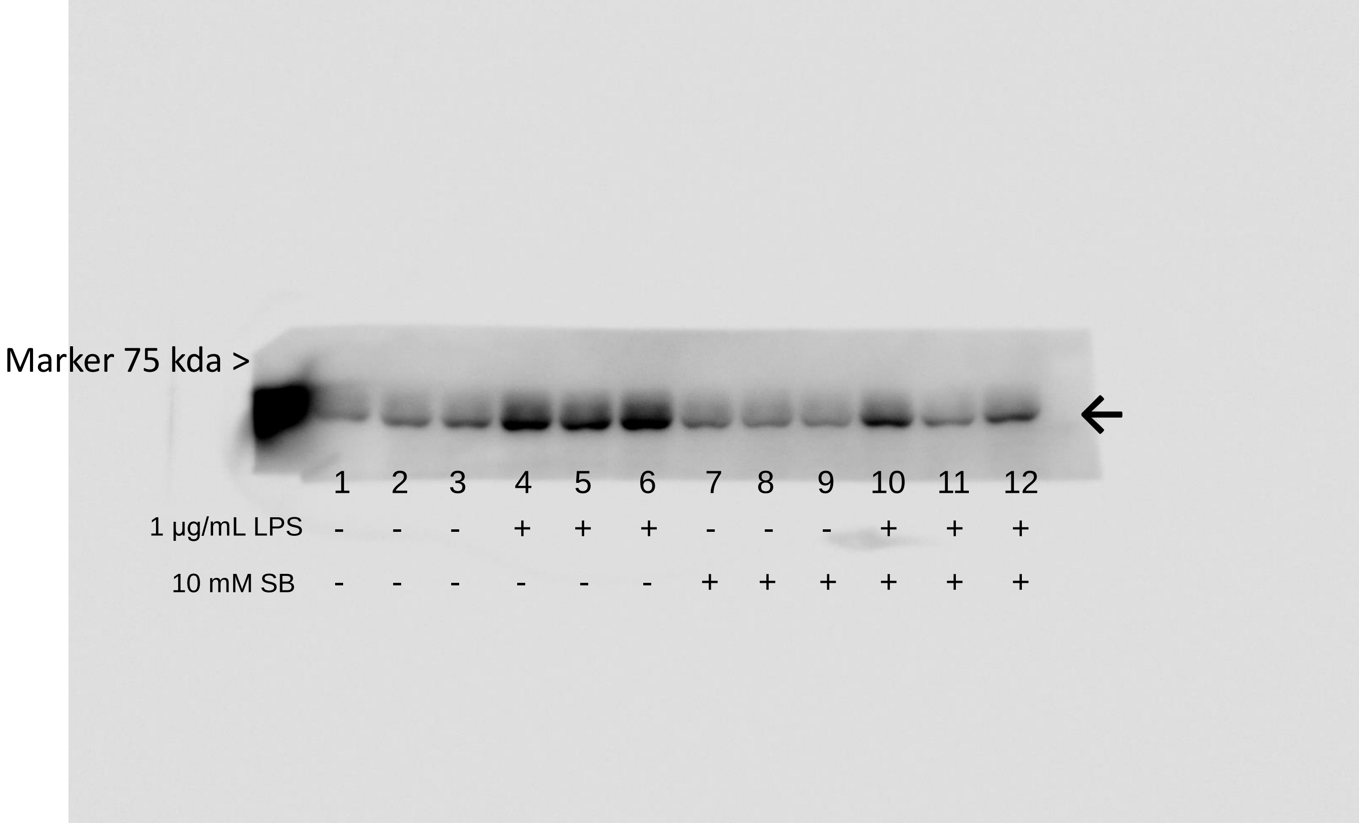

IB: βactin (Figure 7b)

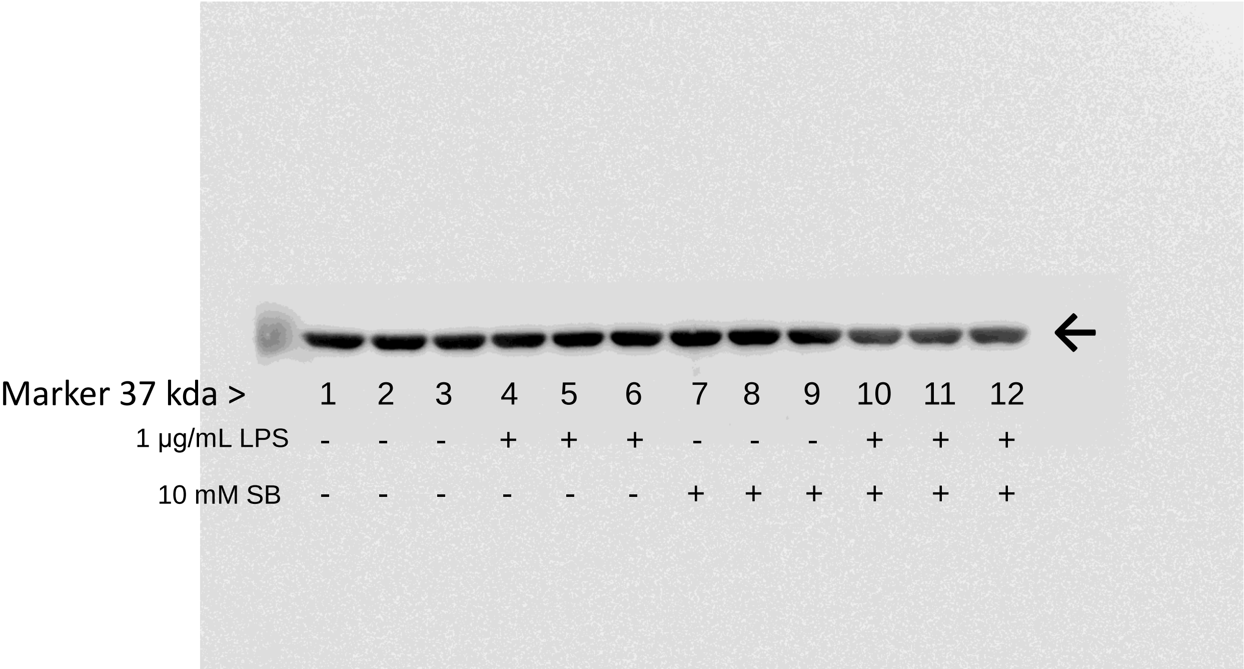

Supplementary Figure S25, Oshima, et al.

IB: RelB (Figure 7b)

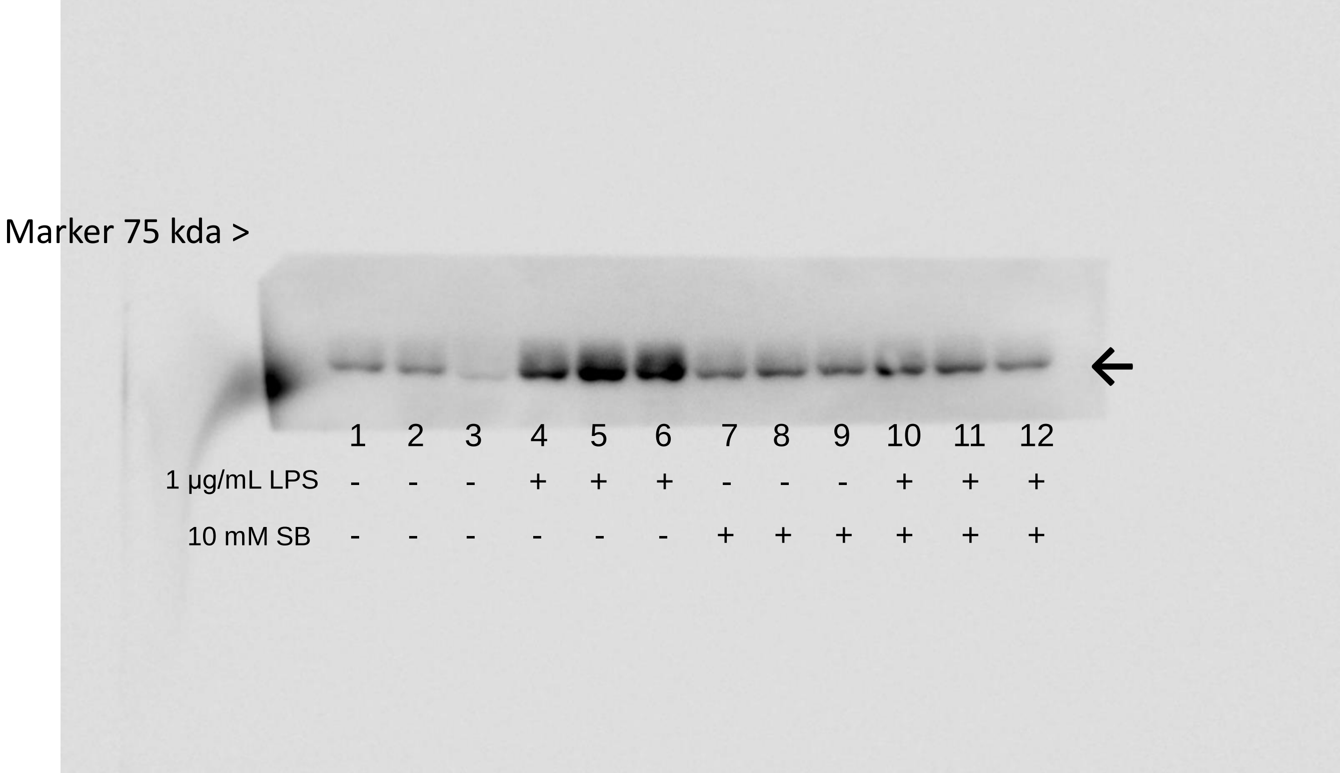

IB: βactin (Figure 7b)

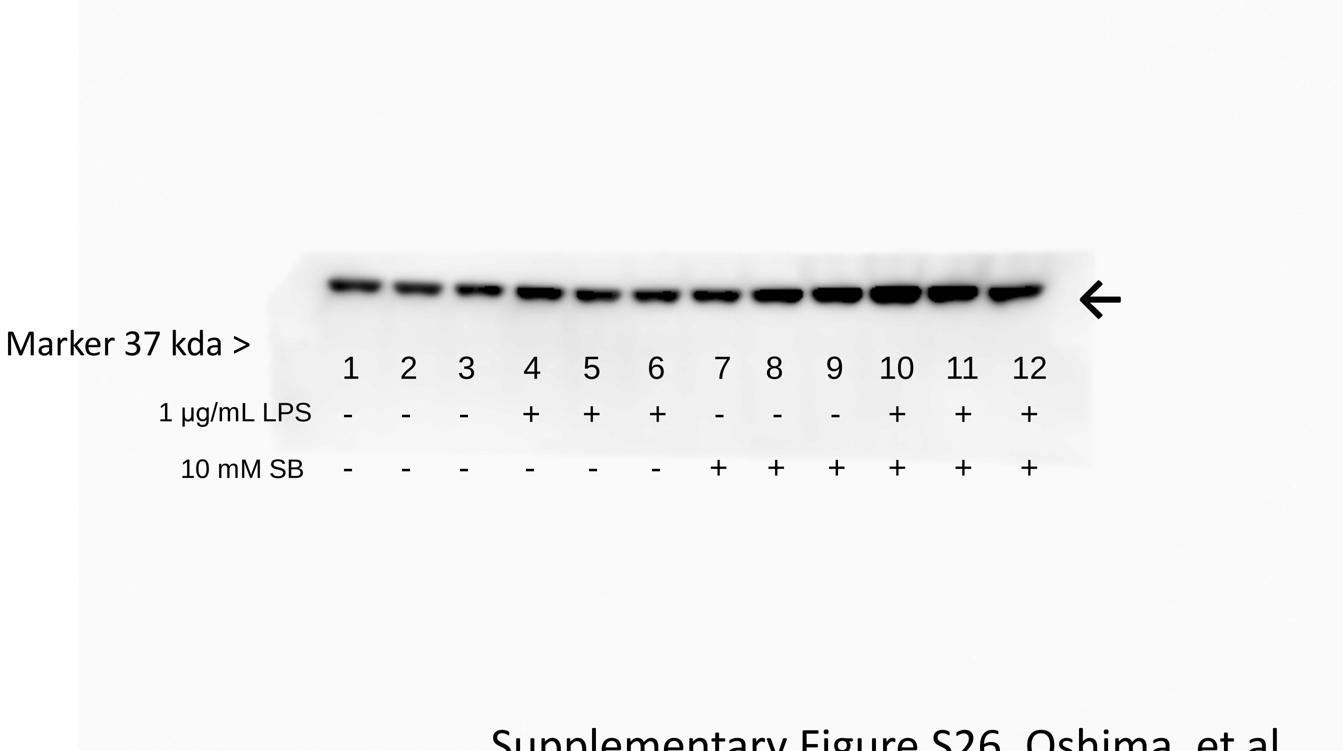

Supplementary Figure S26, Oshima, et al.
